# State Policies Regulating Firearms and Changes in Firearm Mortality

**DOI:** 10.1001/jamanetworkopen.2024.22948

**Published:** 2024-07-31

**Authors:** Terry L. Schell, Rosanna Smart, Matthew Cefalu, Beth Ann Griffin, Andrew R. Morral

**Affiliations:** 1 RAND Corporation, Santa Monica, California; 2 RAND Corporation, Arlington, Virginia

## Abstract

**Importance:**

Despite high social and public health costs of firearm violence in the United States, the effects of many policies designed to reduce firearm mortality remain uncertain.

**Objective:**

To estimate the individual and joint effect sizes of state firearm policies on firearm-related mortality.

**Design, Setting, and Participants:**

In this comparative effectiveness study, bayesian methods were used to model panel data of annual, state-level mortality rates (1979-2019) for all US firearm decedents, with analyses conducted in October 2023.

**Exposures:**

Six classes of firearms policies: background checks, minimum age, waiting periods, child access, concealed carry, and stand-your-ground laws.

**Main Outcome and Measures:**

Primary outcomes (total firearm deaths, firearm homicide deaths, and firearm suicide deaths) were assessed using the National Vital Statistics System. Bayesian estimation was used to estimate the partial association of changes in firearms policies with subsequent changes in firearm mortality.

**Results:**

The estimated effect sizes of individual policies 5 or more years after implementation were generally small in magnitude and had considerable uncertainty. The policy class with the highest probability of reducing firearm deaths was child-access prevention laws, estimated to reduce overall firearm mortality by 6% (80% credible interval [CrI], −2% to −9%). The policy class with the highest probability of increasing firearm deaths was stand-your-ground laws, estimated to increase firearm homicides by 6% (80% CrI, 0% to 13% increase). Estimates of association of implementing multiple firearm restrictions with subsequent changes in firearm mortality yielded larger effect sizes. Moving from the most permissive to most restrictive set of firearm policies was associated with an estimated 20% reduction in firearm deaths (80% CrI, 10% to 28% reduction), with a 0.99 probability of any reductions in firearm death rates.

**Conclusions and Relevance:**

In this comparative effectiveness study of state firearm policies, the joint effect estimates of combinations of firearm laws were calculated, showing that restrictive firearm policies were associated with substantial reductions in firearm mortality. Although policymakers would benefit from knowing the effects of individual policies, the estimated changes in firearm mortality following implementation of individual policies were often small and uncertain.

## Key Points

**Question:**

How has firearm mortality changed following the implementation of several common state policies that regulate firearms?

**Findings:**

This comparative effectiveness study using mortality rates for all US states from 1979 to 2019 found that the estimated effect sizes of firearm regulations on mortality for individual state firearm regulations were often small and uncertain. However, there was a pattern across policies such that the most restrictive set of firearm policies was associated with 20% lower firearm mortality than the most permissive set.

**Meaning:**

The findings of this study suggest that existing state firearm regulations may play a substantial role in mitigating gun violence and might contribute to even greater reductions in mortality if they were more widely adopted.

## Teaser

This comparative effectiveness study estimates the changes in mortality rates associated with common US state firearm policies, including age restrictions, waiting periods, and stand-your-ground laws.

## Introduction

In 2021, an average of 133 people died every day in the United States from firearm-related injuries, and rates of firearm deaths have increased sharply in the past 5 years.^1^ There is an urgent need to identify solutions, including state regulations, that are effective in reducing firearm violence. While certain policies, such as handgun permitting requirements and child access prevention laws, appear likely to reduce some forms of firearm violence,^2,3,4^ much of the scientific literature to date has suffered from weak study designs, low statistical power, and insufficient controls for potential confounding variables.^2,3,4^

State gun policy environments are complex, with multiple statutes—often correlated or intended to be complementary—composing each state’s legal regime. These policy environments, and the outcomes they are intended to produce, may be influenced by key state characteristics, such as state political preferences and firearm ownership rates.^5,6^ This complexity creates challenges for isolating which specific policies may drive effects. Much of the early gun policy evaluation literature examined single laws in isolation,^7^ ignoring these issues. More recent research has explored the effects of multiple gun policies but has generally either (1) included many individual laws in a single model, testing each separately, without consideration of statistical power or the collinearities across policies,^8,9,10^ or (2) used index measures that assume that all firearm policies are equally effective and effects increase linearly with the number of provisions.^11,12,13^ These 2 approaches result in either splitting constructs into a series of conceptually precise policies, where each represents such a narrow fraction of the overall regulatory environment for firearms that it cannot plausibly have large effects, or taking a very broad view in which diverse policies are treated as interchangeable so long as they regulate firearms in some way. In neither case do these methods provide clear information on how firearm policies could be designed to minimize firearm mortality.

The present study aims to better understand the association of state-level firearm policies with subsequent changes in firearm deaths by examining conceptually related policies jointly. Like our earlier efforts,^14^ we model the effect sizes of narrowly defined firearm policies but also estimate and test their joint effect sizes. This approach, which allows the underlying individual policies to have effect sizes that vary in both magnitude and direction, is intended to improve statistical precision in drawing conclusions about the effect sizes of policy classes while preserving theoretical precision in how policies are defined. However, we extend and improve on the earlier literature by expanding the range of policies studied, incorporating a broader set of controls for potential confounding variables, and conducting methodological advancements to better identify causal effects of policies.

## Methods

The study was determined to be exempt from review and from a requirement for informed consent, because it used no identifiable human participant data, by the RAND institutional review board. The study follows good research practices for comparative effectiveness research,^15^ eg, the data sources, modeling methods, primary comparisons, and sensitivity tests were preregistered,^16^ and the data and statistical code are available.^17^ The eAppendix in Supplement 1 provides a detailed discussion of how the current study extends and improves prior published research by the authors.^14^

### Data

Mortality data from 1979 to 2019 come from the National Vital Statistics System, which provides information on coroners’ cause of death determinations for a near-census of deaths in the United States.^1^ Information on the effective dates of firearm laws come from the RAND State Firearm Law Database.^18^ The analyses also include state-level demographic, economic, crime, and gun ownership characteristics; sources for these are described in the eAppendix in Supplement 1.

### Measures

The 3 primary outcomes were annual, state-level rates of firearm-related deaths (total, firearm homicide, firearm suicide). The analyses simultaneously estimated the effect sizes of 10 state firearm policies: background checks for purchase from dealers, background checks for private sales, possession prohibited for individuals younger than 18 years, purchase prohibited for individuals younger than 20 years, 24-hour waiting period, 7-day waiting period, firearm storage restrictions to prevent child access, eliminating discretion in granting concealed carry permits, allowing concealed carry without a permit, and stand-your-ground laws that reduce legal liability associated with use of lethal force in self-defense. Because several of these policies are variations on the same conceptual policy, our primary results group 8 of the individual policies into groups for which we examine the joint effect sizes of 2 policies: background checks for purchase, minimum age requirements for purchase or possession, waiting periods for purchasing a firearm, and removing regulations that restrict the concealed carry of firearms. We also estimate joint effect sizes of these policies for 3 broader policy combinations: purchase and possession of firearms (combining background checks, minimum age, and waiting periods), use and storage of firearms (combining child access, concealed carry, and stand-your-ground policies), and the joint effect size of the most restrictive combination of all 10 underlying policies relative to the least restrictive. The most restrictive combination was defined by the presence of the 4 restrictive classes (background checks, minimum age, waiting periods, and child access) along with the absence of the 2 permissive classes (concealed carry and stand-your-ground). The most permissive is the absence of the 4 restrictive class with the presence of the 2 permissive classes. The particular set of laws included in the study were taken from the RAND Gun Law database and were selected for inclusion in the study because they have the largest number of state policy transitions within the study period of the laws in the database. The eAppendix in Supplement 1 includes detailed information about the definitions used to classify state statutes into broader policy categories and model results for the 10 individual policy types prior to combining them into conceptual policies combinations (eTable 1 and eTable 4 in Supplement 1).

Regression models adjust for year effects and 28 state-level characteristics: (1) 5 covariates considered as serious potential confounding variables that plausibly affect both the passage of firearm policies and firearm mortality rates and (2) 23 state-level characteristics associated with firearm deaths but unlikely to represent potential confounding variables. The 5 potential confounding variables are state household firearm ownership rates, estimated from survey measures of ownership and administrative data sources^19^; political control of the state; the state violent crime rate; the incarceration rate; and state income inequality. The 23 other covariates are largely demographic and economic measures (eTable 2 in Supplement 1).

### Statistical Analysis

To estimate policy effect sizes, we modeled mortality data from 50 states for the years 1981 to 2019 with final analyses conducted in 2023. The regression model used was selected based on Monte Carlo simulations of candidate models to identify the best-performing method for generating accurate estimates of the causal effects of state-level policies on firearm deaths with unbiased estimates of uncertainty.^20,21^

We used a negative binomial regression of each outcome on (1) an offset equal to the natural logarithm of the population in that state-year; (2) effects for each year in the data; (3) first- and second-order autoregressive effects equal to the natural logarithm of the rate of the outcome in the relevant prior year for a given state; (4) state characteristics included as covariates; (5) indicators for each individual policy; and (6) ancillary terms to remove the bias that occurs in autoregressive models of causal effects because such models control for the prior year’s outcome, which is endogenous to the treatment.^21,22^ This model implies that in the absence of a policy change, the year-to-year changes in a state’s firearm mortality rate are expected to be small and will gradually regress toward an expectation set by the characteristics of the state. This is distinct from difference-in-differences approaches, which would assume parallel trends. Furthermore, unlike 2-way fixed-effects approaches, policy effect sizes are not based on differences in outcomes averaged over the full postpolicy and prepolicy periods. The effect estimates for any given policy are identified by the year-to-year changes in the state firearm mortality rate over the first 5 years after it was implemented.

The effects of each policy were parameterized to allow a flexible phase-in over 5 years after implementation, incorporating both an instant effect that occurred at the time of implementation and a linear phase-in term that changes gradually over the 5 years after implementation. Our primary effect estimate combines the instant and phased-in effect, representing the total effect size of the policy with firearm mortality at or beyond the 5-year postimplementation time point. The detailed model specification is in the eAppendix in Supplement 1.

We use bayesian estimation (using Stan version 2.32.2 [Stan Development Team]), which has 3 advantages for our purposes. First, prior simulation studies show that the bayesian estimation of the model we use provides more accurate inference than standard maximum likelihood optimization algorithms.^21^ Second, bayesian methods allow us to estimate the probability that a given policy is associated with an increase or a decrease in firearms deaths. Probabilities like these may be more useful than statistical significance to policymakers deciding whether to support a given firearm policy.^23^ Finally, prior research demonstrates that studies of the effects of state gun policies often lack sufficient statistical power to detect effects of the size likely to be found for these policies, even for an effect size of a magnitude that would be of substantial interest to policymakers (eg, a policy that would reduce firearms deaths by 1000 deaths nationally each year).^20^ Estimates of the magnitude and uncertainty of policy effects using bayesian inference provides a valuable alternative approach to inference that avoids some problems that arise with significance testing with low statistical power.^24^

Priors for each individual policy’s effect estimation were selected such that the total effect size on firearms deaths evaluated 5 years after policy implementation was normally distributed with an equal likelihood that the policy increased vs decreased firearm deaths. When integrated over the coefficients for each policy, the standard deviation of the prior implies a 0.95 probability that the total effect size for each policy on firearm mortality falls between incidence rate ratios (IRRs) of 0.82 and 1.22, implying that it is unlikely any single policy would change firearm mortality rates more than approximately 20%. The selection of this prior is based on an earlier survey of gun policy experts, which showed this range of expected gun policy effect sizes.^25^ Coefficients for the 23 covariates judged as unlikely to represent confounding variables were estimated with a regularizing prior (bayesian lasso).^26^ eTables 5 to 9 in Supplement 1 contain sensitivity tests and related discussions including results using uninformative priors.

## Results

The estimated model (eTable 3 in Supplement 1) provided an excellent fit to the data, with squared correlations between the posterior median predicted death rate and the observed death rate across all data points of 0.95, 0.93, and 0.95, for total firearm deaths, firearm suicides, and firearm homicides, respectively. Table 1 provides model results for the effect estimates for the 6 classes of firearms policies on the 3 primary outcomes.

**Table 1.** Effect Sizes of Firearm Policies on State Firearm Death Rates From the Fifth Year After Implementation^a^

Law class	Posterior median IRR	80% CrI	95% CrI	Posterior probability of reduced deaths
**Total firearm deaths**				
Background checks	1.02	0.97-1.08	0.94-1.11	0.31
Minimum age	0.95	0.89-1.01	0.86-1.04	0.87
Waiting periods	0.95	0.90-1.01	0.88-1.04	0.86
Child access	0.94	0.91-0.98	0.89-1.00	0.98
Concealed carry	1.05	0.99-1.11	0.96-1.15	0.16
Stand-your-ground	1.03	0.99-1.07	0.98-1.09	0.14
**Firearm suicides**				
Background checks	0.96	0.91-1.01	0.89-1.04	0.84
Minimum age	0.94	0.89-0.99	0.86-1.02	0.93
Waiting periods	0.98	0.93-1.03	0.91-1.06	0.71
Child access	0.98	0.94-1.01	0.93-1.03	0.80
Concealed carry	1.04	0.98-1.10	0.95-1.13	0.19
Stand-your-ground	1.02	0.99-1.05	0.97-1.07	0.22
**Firearm homicides**				
Background checks	1.07	0.98-1.17	0.93-1.23	0.17
Minimum age	0.96	0.87-1.06	0.83-1.11	0.71
Waiting periods	0.99	0.91-1.08	0.87-1.14	0.53
Child access	0.91	0.86-0.97	0.83-1.00	0.97
Concealed carry	1.08	0.98-1.19	0.94-1.25	0.15
Stand-your-ground	1.06	1.00-1.13	0.97-1.16	0.09

Abbreviations: CrI, credible interval; IRR, incidence rate ratio.

^a^ Concealed carry and stand-your-ground law classes are coded as reductions in firearm restrictions; all other policies increase restrictiveness.

Each individual policy class had a relatively small effect size, most of which did not clearly determine whether the policy would increase or decrease deaths. The exceptions were child access prevention policies, which were associated with a 0.98 and 0.97 probability of reductions in total firearm deaths and firearm homicides, respectively, given the data, the model, and the priors. Additionally, stand-your-ground policies were associated with a 0.91 probability of increases in firearm homicides. The expected magnitude of these policies’ effect sizes was moderate, ie, a 6% to 7% change in deaths. For child access prevention, the estimated effect size on total firearm deaths increased over the 5-year period, while the effect size for stand-your-ground laws was stable (eFigure 1 in Supplement 1).

Despite the relatively small and uncertain effect sizes for individual policies, the joint effect sizes of conceptually related policies were often much larger (Table 2; eFigure 2 in Supplement 1). Restrictive combinations of use and storage policies, for instance, were estimated to have a 0.99 probability of reductions in total firearm deaths given the data, the model, and the priors. The estimated effect size after 5 years was a 13% reduction (IRR, 0.87; 80% CrI, 0.80-0.94). There was less certainty about the effect sizes for policies restricting the purchase and possession of firearms, which were estimated to have a 0.89 probability of reductions in firearm deaths (IRR, 0.92; 80% CrI, 0.85-1.00). Combining all policies provided evidence that the implementation of firearm restrictions was associated with subsequent reductions in firearm mortality (Figure); comparing a policy regime with all firearm restrictions with a regime with none was associated with 20% lower firearm mortality (IRR, 0.80; 80% CrI, 0.72-0.90) and a 0.99 probability of reductions in firearms deaths, given the data, the model, and the priors. A reduction in risk of this magnitude would imply approximately 70 000 fewer deaths between 2010 and 2020.

**Table 2.** Effect Sizes of Policy Combinations on State Firearm Death Rates From the Fifth Year After Implementation^a^

Law class	Posterior median IRR	80% CrI	95% CrI	Posterior probability of reduced deaths
**Total firearm deaths**				
Purchase and possession	0.92	0.85-1.00	0.81-1.05	0.89
Use and storage	0.87	0.80-0.94	0.77-0.98	0.99
Most restrictive	0.80	0.72-0.90	0.67-0.96	0.99
**Firearm suicides**				
Purchase and possession	0.88	0.82-0.95	0.78-0.99	0.98
Use and storage	0.92	0.86-0.99	0.82-1.03	0.92
Most restrictive	0.81	0.73-0.90	0.69-0.95	0.99
**Firearm homicides**				
Purchase and possession	1.02	0.90-1.17	0.83-1.25	0.41
Use and storage	0.80	0.70-0.90	0.66-0.96	0.99
Most restrictive	0.81	0.68-0.97	0.62-1.07	0.93

Abbreviations: CrI, credible interval; IRR, incidence rate ratio.

^a^ The purchase and possession law class estimates the combined effect size of restrictions on firearms through background check, minimum age requirement, and waiting period laws; the use and storage class estimates the combined effect size of restrictions through child access, concealed carry, and stand-your-ground laws. The most restrictive class estimates the combined effect size of the presence of the 4 restrictive classes and the absence of the 2 permissive classes.

**Figure.**
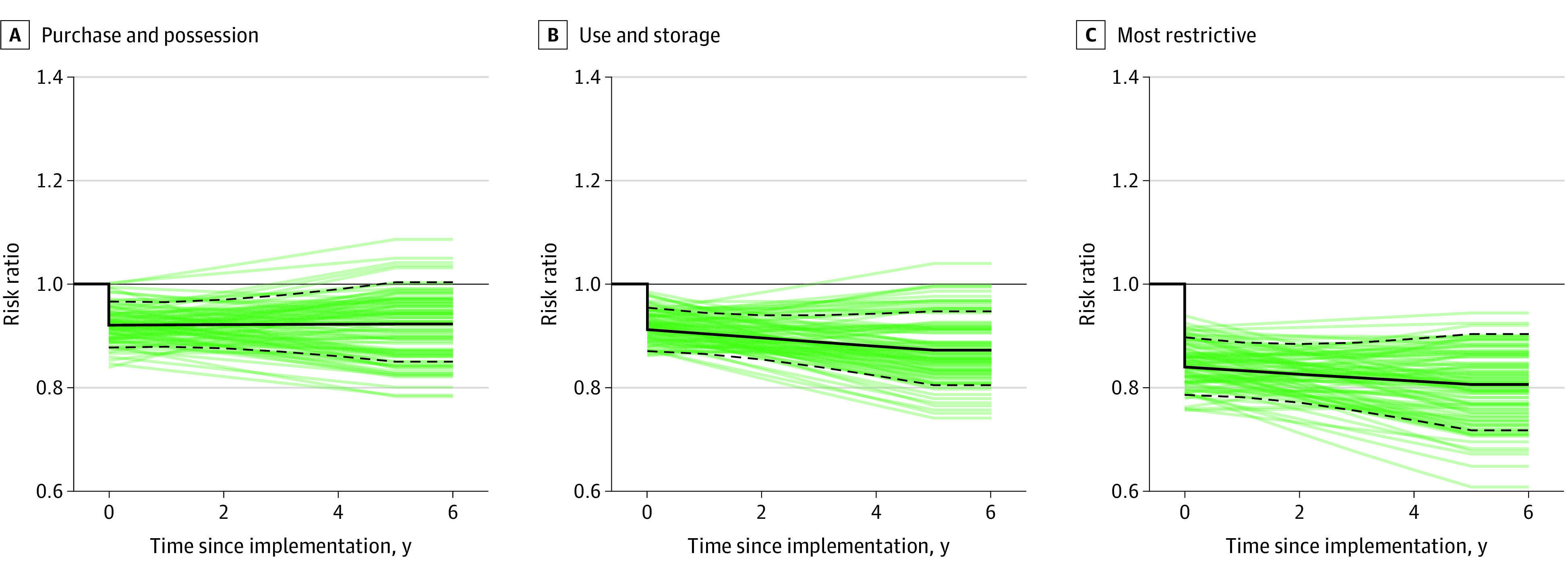
Effect Sizes of Combinations of Firearm Regulations on Total Firearm Deaths, by Time Since Implementation The posterior median (solid black line), 80% credible interval (dashed black lines), and 100 samples (green lines) from the posterior distribution are plotted.

Effect sizes of the restrictive combination of policies for firearm suicide (IRR, 0.81; 80% CrI, 0.73-0.90) and firearm homicide (IRR, 0.81; 80% CrI, 0.68-0.97) were similar in magnitude, although there was more certainty in the direction for firearm suicide deaths than for firearm homicide deaths (probabilities of 0.99 and 0.93, respectively). Furthermore, there was some evidence that different types of policies matter for the different outcomes. For firearm suicide, the reduction associated with the overall restrictive policy regime was from both restrictions on purchase and possession (IRR, 0.88; 80% CrI, 0.82-0.95) and restrictions on use and storage (IRR, 0.92; 80% CrI, 0.86-0.99). For firearm homicide, however, the overall estimated effect size of a restrictive policy environment was entirely from the restrictions on use and storage (IRR, 0.80; 80% CrI, 0.70-0.90).

Policy effect sizes for firearm homicides and suicides did not appear to be meaningfully offset by compensatory effects on nonfirearm deaths (eTable 8 in Supplement 1). Instead, policy effect sizes for all homicides and all suicides were generally similar but somewhat smaller in magnitude than for the firearm-specific outcomes.

## Discussion

Reviews of research on the effects of firearm policies have noted that effect estimates of such policies are often small or provide inconclusive evidence for the direction of the effects.^3^ Similarly, when looking at policies individually, the current study found few individual policies showed evidence of a reliable association with subsequent changes in total firearm mortality. Yet, estimating effect sizes jointly across policies, we found that a restrictive firearm policy regime was associated with 20% fewer firearm deaths than a permissive regime. This is a much larger effect size than any policy considered alone, with higher confidence in the direction of those joint effect sizes. Although there was considerable uncertainty around which specific policies were driving that large effect size, this finding provides evidence that this combination of firearm restrictions was associated with substantial reductions in firearm mortality in the 5 years after implementation.

Dividing the regulatory environment for firearms into small, conceptually precise policy types that are tested individually is theoretically appealing but may result in poorly estimated causal effects. These imprecise and nonsignificant results may be incorrectly interpreted as evidence that the policies are not effective for reducing gun violence. Estimating the joint effect sizes of laws commonly implemented together may provide a more useful approach for understanding how firearm policy affects firearm violence.

Our results are broadly consistent with other research that has estimated the joint effects of multiple types of firearm policies using different methods, such as using counts of policies as an index indicating the restrictiveness of a state’s policy regime.^11,12,13^ Those studies also found some evidence that firearm restrictions were associated with better mortality outcomes, but our current approach to aggregating policy effect sizes has several advantages over methods using policy index measures. Our approach does not require the strong assumption that all policies being jointly estimated have identical effect sizes on each outcome. To the extent that assumption is wrong, policy index measures are likely to misestimate the effect sizes of the studied laws or will fail to reliably find any effect.

Firearm violence is affected by cultural, economic, law enforcement, and mental health factors in addition to the firearm regulations we examined here. Our finding that most of these individual state-level firearm policies have relatively modest and uncertain effect sizes reflects that each firearm policy is a small component of a complex system shaping firearm violence. However, we found that combinations of the studied policies were reliably associated with substantial shifts in firearm mortality.

### Limitations

There are also several limitations to the current study that qualify our conclusions. The results of this study describe the probability that studied firearm laws are associated with reductions in firearm deaths, given the data, the model, and the priors. These results do not capture uncertainties in the data or model. We may have underestimated uncertainty if the data had substantial measurement error or if the model did not adequately capture key features of the true data-generating mechanism. For example, if our measures of potential confounds had high measurement error, we may have failed to remove all bias in the causal effect estimates due to that confounding. Similarly, the model itself may have omitted variables that have a causal effect on both the probability of adopting these polices and on changes in future mortality rates conditioned on the other covariates in the model. To the extent the model omitted such variables, the effect estimates may overestimate or underestimate the true causal effect. To partially address these concerns, we conducted several sensitivity tests in the eAppendix in Supplement 1 that changed the model or used alternative methods to identify causal effects, all of which showed a robust pattern of findings.

Similarly, the joint effect sizes we estimated rest on a model assumption that the risk ratio for a given policy is constant and does not change based on what other policies are present, what year it was implemented, or the values of the covariates. If the true causal effects are heterogeneous, these averages may not accurately reflect the effects of a policy implemented in a specific state at a specific time. Because there are relatively few policy changes across the 50 states and high correlation in the policies selected by states, testing for the presence of heterogenous effects is not feasible. Additional discussion on this point appears in the eAppendix in Supplement 1. For example, while one might hypothesize that permitless carry laws have different effects in states with stand-your-ground laws, we cannot test such an interaction because almost all permitless carry laws were adopted in states that already had stand-your-ground laws.

The method we used relied on policy variation across states for identification. While some policies we evaluated have been enacted at the federal level, our method cannot provide effect estimates for federal policies. The effects of national laws could be different than the effects of similar policies implemented by individual states if, for example, it is easier to evade firearm purchase restrictions when they are not enforced in neighboring states. Similarly, we are limited to studying policies that have been implemented in several states since 1982, which may not capture some of the most innovative or effective strategies that have been proposed to mitigate firearm violence.

## Conclusions

In this study, the most restrictive firearms policy regime was associated with a 20% reduction in firearm deaths, equivalent to preventing approximately 70 000 deaths over the decade ending in 2020. Existing state firearm regulations may already play a substantial role in mitigating gun violence and might contribute to even greater reductions in mortality if they were more widely adopted.

## Notes

Supplement 1. **eAppendix.** Supplementary Methods, Results, and Discussion **eTable 1.** Years of Implementation for Firearms Policies **eTable 2.** State Characteristics Included as Covariates **eTable 3.** Posterior Medians and 95% Credible Intervals for Model Parameters by Type of Firearm Death **eTable 4.** Effect Sizes of Law Subclasses on State Firearm Death Rates in the Fifth Year After Implementation, for Law Classes With Multiple Levels **eTable 5.** Effect Sizes of State Laws on Firearm Death Rates in the Fifth Year After Implementation for Those Firearm Restrictions Implemented Nationally in 1994 **eTable 6.** Effect Sizes of Law Classes on State Firearm Death Rates in the Fifth Year After Implementation Estimated Using Uninformative Priors **eTable 7.** Effect Sizes of Law Classes on State Firearm Death Rates in the Fifth Year After Implementation Estimated Without Additional Lagged Policy Spline **eTable 8.** Effect Sizes of Law Classes on Total Suicide and Total Homicide Death Rates in the Fifth Year After Implementation **eTable 9.** Separate Effect Estimates for Each Policy on Total Firearm Deaths Using the Callaway and Sant’Anna (2021) Staggered DiD Method **eFigure 1.** Effect Estimates of 6 Classes of Firearm Regulations Over Time, by Type of Firearm Death **eFigure 2.** Effect Estimates of Combinations of Firearm Regulations Over Time, by Type of Firearm Death
eReferences.

Supplement 2.
Data Sharing Statement
