# Supplementary material for: State Policies Regulating Firearms and Changes in Firearm Mortality
Source: JAMA Netw Open. 2024 Jul 31;7(7):e2422948. doi: 10.1001/jamanetworkopen.2024.22948 (PMC11292452; doi:10.1001/jamanetworkopen.2024.22948)
Supplement: Supplement 1. — eAppendix. Supplementary Methods, Results, and Discussion eTable 1. Years of Implementation for Firearms Policies eTable 2. State Characteristics Included as Covariates eTable 3. Posterior Medians and 95% Credible Intervals for Model Parameters by Type of Firearm Death eTable 4. Effect Sizes of Law Subclasses on State Firearm Death Rates in the Fifth Year After Implementation, for Law Classes With Multiple Levels eTable 5. Effect Sizes of State Laws on Firearm Death Rates in the Fifth Year After Implementation for Those Firearm Restrictions Implemented Nationally in 1994 eTable 6. Effect Sizes of Law Classes on State Firearm Death Rates in the Fifth Year After Implementation Estimated Using Uninformative Priors eTable 7. Effect Sizes of Law Classes on State Firearm Death Rates in the Fifth Year After Implementation Estimated Without Additional Lagged Policy Spline eTable 8. Effect Sizes of Law Classes on Total Suicide and Total Homicide Death Rates in the Fifth Year After Implementation eTable 9. Separate Effect Estimates for Each Policy on Total Firearm Deaths Using the Callaway and Sant’Anna (2021) Staggered DiD Method eFigure 1. Effect Estimates of 6 Classes of Firearm Regulations Over Time, by Type of Firearm Death eFigure 2. Effect Estimates of Combinations of Firearm Regulations Over Time, by Type of Firearm Death eReferences. [file jamanetwopen-e2422948-s001.pdf]

Supplemental Online Content

Schell TL, Smart R, Cefalu M, Griffin BA, Morral AR. State policies regulating firearms and changes in firearm mortality. *JAMA Netw Open.* 2024;7(7):e2422948. doi:10.1001/jamanetworkopen.2024.22948

eAppendix. Supplementary Methods, Results, and Discussion

eTable 1. Years of Implementation for Firearms Policies

eTable 2. State Characteristics Included as Covariates

eTable 3. Posterior Medians and 95% Credible Intervals for Model Parameters by Type of Firearm Death

eTable 4. Effect Sizes of Law Subclasses on State Firearm Death Rates in the Fifth Year After Implementation, for Law Classes With Multiple Levels

eTable 5. Effect Sizes of State Laws on Firearm Death Rates in the Fifth Year After Implementation for Those Firearm Restrictions Implemented Nationally in 1994

eTable 6. Effect Sizes of Law Classes on State Firearm Death Rates in the Fifth Year After Implementation Estimated Using Uninformative Priors

eTable 7. Effect Sizes of Law Classes on State Firearm Death Rates in the Fifth Year After Implementation Estimated Without Additional Lagged Policy Spline

eTable 8. Effect Sizes of Law Classes on Total Suicide and Total Homicide Death Rates in the Fifth Year After Implementation

eTable 9. Separate Effect Estimates for Each Policy on Total Firearm Deaths using the Callaway and Sant'Anna (2021) Staggered DiD Method

eFigure 1. Effect Estimates of 6 Classes of Firearm Regulations Over Time, by Type of Firearm Death

eFigure 2. Effect Estimates of Combinations of Firearm Regulations Over Time, by Type of Firearm Death

eReferences.

This supplemental material has been provided by the authors to give readers additional information about their work.

© 2024 Schell TL et al. *JAMA Network Open.*

eAppendix. Supplementary Methods, Results, and Discussion

State Laws. We estimate effects for ten separate laws or policies that fall into several broader classes. For all of these laws the implementation dates were drawn from the RAND State Firearm Law Database (Cherney et al., 2020). eTable 1 contains data on the years over which each policy was in effect within each state. More detail on the specific months of implementation and the law citations are available in the RAND database. This particular set of laws was preregistered and were selected for inclusion because they have the largest number of state policy transitions within the study period of those laws in the database. In general, the database codes laws based on their effect on policy rather than how the law describes itself. For example, if a state's "firearm permit" law requires, among other things, a background check before any purchases of handgun we code the law as implementing a universal background check policy.

The study evaluated six law classes that regulate the purchase or possession firearms, this includes:

- Two levels of laws regulating background checks for the purchase of firearms:
  - laws requiring federal firearms licensed dealers to conduct background checks (DBC).
  - laws requiring all handgun sales to include a background check (UBC).
- Two levels of laws regulating minimum age requirements to possess or purchase a handgun:
  - laws setting a minimum age of possession to 18 years or older (MA18).
  - laws setting a minimum age of purchase to 20 years or older (MA20).
- Two levels of laws regulating waiting periods between handgun purchase from a dealer and full possession:
  - laws requiring at least 24-hours waiting period (WP1).
  - laws requiring at least a 7-day waiting period (WP7).

We also included four law classes that regulate the use or storage of firearms:

- Two levels of laws that reduce restrictions on carrying a concealed weapon relative to jurisdictions that require concealed carry permits and allow law enforcement discretion in issuing permits.
  - Shall-issue laws that do not allow law enforcement discretion to deny the right to carry a concealed weapon to all those who meet its permit requirements (SI)
  - Laws that allow concealed carry without a permit for anyone who can legally possess the weapon (PC).
- Stand your ground laws (SYG) that permit the use of lethal force for self-defense outside of the defender's home or vehicle, even when a retreat from danger would have been possible. Following the RAND law database, case law is not classified as a stand-your-ground law in the absence of a state statue granting those rights.
- Child access prevention laws (CAP) that specify either civil or criminal penalties for storing a handgun in a manner that allows access by a minor.

Our primary interest is to understand which classes of laws are associated with changes in firearm deaths in the 5-years immediately after their implementation. For this reason, we compute effect sizes for combinations of individual laws. These combinations reflect the fact that several classes of gun laws are conceptually related and empirically correlated. The analytic approach allows the research to answer broad questions about which types of regulations have either beneficial or harmful effects without requiring assumptions that all laws within a class have identical effects. For each of these classes of laws listed below, we compare the most restrictive combination of laws in the class to the most permissive combination.

- The *Background Check* effect compares having universal background checks from dealer and nondealer sellers (DBC + UBC) relative to having no background check requirement.
- The *Minimum Age* effect compares having no handgun possession until 18+ and no handgun purchase until 20+ (MA18 + MA20), relative to allowing 17-year-olds to purchase and possess a handgun.
- The *Waiting Period* effect compares having a waiting period of 7+ days (WP1 + WP7) for a handgun purchased from a dealer relative to allowing immediate possession following purchase.
- The *Concealed Carry* effect compares permissive concealed carry regulations (SI + PC) to more restrictive regulations that give law enforcement discretion in issuing permits.

- The *Purchase or Possession* effect compares a restrictive condition in which there are universal background checks, no handgun possession unless 18, no handgun purchase unless 20, and a seven-day waiting period (DBC + UBC + MA18 + MA20 + WP1 + WP7) relative to a permissive condition in which there are no required background checks, waiting periods, or restrictions on handgun possession for those under 18.
- The *Use and Storage* effect compares a restrictive condition in which there is law enforcement discretion in determining who can carry a concealed weapon, a duty to retreat from danger when possible before using lethal force outside the home, and penalties for storing a firearm in a way that allowed access by a minor (CAP) to a permissive condition in which everyone who can legally possess a firearm can carry a concealed weapon, one can legally use lethal force before exercising safe retreat options, and there are no penalties for allowing firearm access to a minor (SI + PC + SYG)
- The *Overall Restrictions* effect compares a condition with the most restrictive combination of laws (DBC + UBC + MA18 + MA20 + WP1 + WP7 + CAP) relative to one with the most permissive (SI + PC + SYG).

Covariates. The model includes year effects as well as 28 covariates which assess state characteristics in each year. These are divided into two broad classes (see eTable 2). Five of the covariates were identified by the research team as potential serious confounds based on our understanding of the existing theory and literature; these represent factors that could affect both (a) whether or when states altered their firearm regulations, and (b) the rate of firearm deaths in the state. For example, states with Democratic control of the government might be more likely to implement restrictions on firearms but might also make a variety of other policy choices that could affect firearm death rates. Similarly states with high rates of gun ownership might be less likely to implement restrictions on firearms and also have more firearm deaths for reasons unrelated to those regulations.

The remaining 23 state characteristics were seen as unlikely to be substantial confounds but which might be associated with firearm deaths and represent sources of exogenous variation, variation that might lead to less accurate causal effect estimates if not accounted for in the models. As discussed in the model section below, this class of covariates was included in the model with regularization. Their coefficients were shrunk toward zero such that they were included in the model only to the extent their inclusion improved prediction of firearm deaths. To be clear, both sets of covariates were included as control variables within the model, but the variables viewed as the most serious potential confounds were included as fixed effects, while the remainder are included as random effects.

In addition to classifying whether each covariate is a likely confound, the research team classified whether they were potential colliders, variables that may be affected by either the firearm policies of interest or by firearm mortality rates. Including such variables as covariates in the model will bias causal effect estimates (Pearl, 2009). The research team identified 4 variables they suspected could be affected by either the laws of interest or by firearm death rates (in addition to possibly affecting those same constructs). These variables are marked with an asterisk in eTable 2. To mitigate possible collider bias while still controlling for these constructs as possible confounds we lagged these variables by five years in the analysis; that is, when predicting deaths in 2000 we use the 1995 value of the covariate. This lagging in time reduces possible collider bias by using a version of the variable that temporally precedes the outcome it is predicting, as well as preceding the policy indicators that are predicting that same outcome. This increases the likelihood that the empirical association of the analytic variable with both the outcome and the treatments reflects the causal effects of that potential confound (e.g., gun ownership rates), rather than the association due to the causal effect of either the gun policies or firearm mortality on the potential confound, such as firearm ownership. This allows us to control for constructs that are likely confounds in a way that minimizes estimation biases that could be caused by possible reciprocal causal relationships between the construct and either the firearm policies or firearm mortality rates. These decisions were all included in our preregistration materials before estimating policy effect.

The state characteristics are primarily standard measures from publicly available, government sources (Census Bureau, Federal Bureau of Investigations, National Institute on Alcohol Abuse and Alcoholism). The four exceptions are: *Household gun ownership rate*, which is taken from Schell et al, (2020a); *Political control of state*, which is derived as the proportion of legislative veto points controlled by Republicans using data from the National Conference of State Legislatures; *Proportion of income for top 10%* which is a measure of income inequality included in the World Inequality Database (Alvaredo, et al.,

2020), and *Wave of U.S. expansion* which is a three-level measure of how recently a state was admitted to the union: (1) Original 13 states, (2) admitted before 1865, and (3) admitted after 1865.

Prior to analysis, these variables were transformed to address undesirable distributional properties. In a few cases we imputed missing state-year values using linear interpolation between the prior-year and subsequent-year values for that state. For a few predictors with extreme outliers, we applied transformations to limit the influence of outlier values: we applied the minimal power transformation (e.g., square root) that ensured all values were within four standard deviations of the mean. We reduced collinearity between these state characteristics and the year effects included in the model by removing the national trend from each. Finally, time-varying covariates were standardized to mean = 0, standard deviation = 1.

Statistical Model. The selection of the model was based on a series of simulation studies to identify the best model for analyzing state-level firearm death data (Schell, Griffin, and Morral, 2018; Cefalu et al., 2021). These studies demonstrated that a particular autoregressive model yielded accurate type I error (i.e., unbiased standard errors), the highest statistical power (i.e., lowest error of the estimate), and minimal bias. These models substantially outperformed more common models, such as two-way fixed effects, with respect to accurate standard errors, low variance/accurate estimates, and the sensitivity of results to included covariates. However, to get the benefits of any autoregressive model one needs to address bias in the magnitude of the effect size estimate that occurs because the outcome at the prior time period is endogenous to the treatment of interest. While we have demonstrated three methods to do this (Schell, Griffin, and Morral, 2018; Schell et al., 2020b; Cefalu et al., 2021), the current study uses the method demonstrated in Cefalu et al. (2021). Of the methods we have investigated, this is the most accurate and flexible approach to correct for the biases in the effect sizes of autoregressive models of count outcomes.

For simplicity, we present a version of the model below estimating the effect of a single law, although our final model included indicators for all ten laws so that all effects control for the possible effects of correlated laws. For a specific mortality outcome and a single law we have a model of the following form:

$$Y_t \sim NegativeBinomial(\mu_t, 1/\phi)$$
$$\ln(\mu_t) = \ln(N_t) + \delta_1 \ln(Y_{t-1}/N_{t-1}) + \delta_2 \ln(Y_{t-2}/N_{t-2}) + \beta_1 PI_t + \beta_2 PS_t + \beta_3 PL_t + \boldsymbol{\gamma X_t} + \boldsymbol{\nu U_t} + \alpha$$
$$- \delta_1\beta_1 PI_{t-1} - \delta_1\beta_2 PS_{t-1} - \delta_1\beta_3 PL_{t-1} - \delta_2\beta_1 PI_{t-2} - \delta_2\beta_2 PS_{t-2} - \delta_2\beta_3 PL_{t-2} \quad (1)$$

Where Y_t is the number of firearm deaths in a given state in year t predicted in a log-link, negative binomial model from:

- the population in that state-year, N_t, which is logged and used as an offset;
- second-order autoregressive predictors, $\ln(Y_{t-1}/N_{t-1})$ and $\ln(Y_{t-2}/N_{t-2})$;
- a dichotomous indicator of when a given policy is in effect, PI;
- a time spline, PS, that increases linearly from 0 to 1 for five-years following that policy's implementation and continues at 1 thereafter;
- a time spline, PL, that increases linearly from 0 beginning five-years after that policy's implementation, reaching 1 at 10-years post-implementation and continuing at 1 thereafter (see below for a discussion of this term's role in the model);
- a vector of covariates that we judged to be possible confounds, $\boldsymbol{X}$, including indicators of year as well as the variables indicated in eTable 2;
- a vector of covariates that we judged unlikely to be confounds, $\boldsymbol{U}$; and
- a constant, α.

The terms in the second row of equation 1 include no additional parameters, but are included to debias the causal effect estimates by removing the portion of the prediction at time t that is endogenous to the policy at times t-1 and t-2 as a result of including Y_{t-1} and Y_{t-2} as predictors in the model even though those variables also depend on β_1, β_2, and β_3 (see Cefalu et al., 2021).

The model was estimated using Bayesian methods in Stan 2.22. The priors used were:

$\delta_1 \sim$ N(mean = 0.5, sd = 1) The first order autoregressive coefficient is likely to fall between zero and one. This was selected to be a minimally informative prior

© 2024 Schell TL et al. *JAMA Network Open.*

δ_2 ~ N(mean = 0, sd = 1) The second order autoregressive coefficient is likely to fall between -1 and 1. This was selected to be a minimally informative prior.

β_1, β_2, β_3 ~ N(mean = 0, sd = 0.071) with policy variables in the range of 0/1. The standard deviation of the total policy effect at five years post-implementation expected with this prior ($\beta_1 + \beta_2$) is 0.071*sqrt(2) = 0.10 for total firearm deaths and firearm homicides. For firearm suicides we use N(mean = 0, sd = 0.064) which corresponds to a total effect standard deviation of 0.09 across both parameters. See discussion below.

γ ~ N(mean = 0, sd = 0.1) for standardized, continuous X's and N(mean = 0, sd = 0.2) for the 0/1 year indicators. These are weakly informative priors within this model because these outcomes are highly stable year to year and the model conditions on prior years' mortality rates.

ν ~ Laplace(mean = 0, sd = Θ) We use a Bayesian LASSO to regularize covariates in X. ν is the parameter that controls the extent of regularization.

Θ ~ Half-cauchy(mean = 0, sd = 1) This is a relatively uninformative prior on the regularizing hyperprior.

ϕ ~ Half-normal(mean = 0, sd = 0.1) This is an uninformative prior on the inverse of the negative binomial overdispersion parameter.

The priors for each law's total effect at 5-years post implementation (i.e., $\beta_1 + \beta_2$ for a given law) were selected such that the total effect on ln(firearm deaths) is normally distributed and centered on no effect (i.e., equal likelihood that the law increased versus decreased firearm deaths). When integrated over the coefficients for each law, the standard deviation of the prior implies a 0.95 probability that the total effect size for each law on firearm mortality falls between a ln(IRR) of -0.2 and 0.2 (corresponding to IRRs of 0.82 and 1.22). This is an informative prior. The selection of this prior is based on an earlier survey of gun policy experts which showed expected gun policy effect sizes in the range described by this prior (Smart, Morral and Schell, 2020). Specifically, from that expert survey, we determined that expert expectations of the effect sizes for a wide range of gun laws (expressed on a log IRR scale) deviate from zero by an average of 0.10 when predicting total firearm deaths, 0.10 for firearm homicide, and 0.09 for firearm suicide.

To correctly parametrize the effect of each law class, we must make an assumption about how long laws take to achieve their full effects. For example, a law may make it easier to get a concealed carry permit immediately on its implementation date, but it may take years for the proportion of the population with such permits to increase to a stable level. In our models, we have chosen the effect during the 5th year post-implementation as our primary timepoint for the estimation of the effects. The specific coding of the laws we used will allow for a nonlinear phase-in of the effect over the first five years after implementation. Specifically, each of the 10 law indicators has one function, PI, that allows for an immediate change after implementation of the policy, and a second function, PS, that phases in slowly over five years. The total effect of each law indicator is then assessed by combining these separate effects.

The primary specification also includes a third function, PL, of each law that is designed to ensure that our estimates of law effects are not influenced by changes in mortality rates that occur a decade or more after their implementation. The coefficient on this function is not included in the law effect estimates, and it is structured so that any changes in mortality rates that occur more than 10 years after law implementation do not influence our estimate of policy effects. Changes in mortality rates that occur between 5 and 10 years after law implementation have progressively less influence on our estimate of policy effects. This model term is included to ensure that the changes in mortality rates used to identify causal effects occurred near the time of implementation. It also reduces concerns about the inclusion of several covariates that may be endogenous to gun deaths or gun policy. As discussed earlier, these covariates were lagged by five years prior to inclusion in the model but could be seen as collider variables beyond that 5-year period. The inclusion of the PL spline helps to ensure that our effect estimates are not biased by these colliders. A version of the effect estimated when dropping the PL spline from the model is included as a sensitivity test later in this appendix (eTable 7).

The outcome is modelled as discrete-time, based on calendar year, although data on the implementation of various laws is known with greater precision than yearly. To take advantage of that precision, we first compute monthly values of the various treatment indicators then the annual values used in the model (PI, PS, PL) are computed as the average of the monthly spline value over each calendar year.

Thus, the nominal 5-year phase in period extends across 6 calendar years for laws not implemented on January 1st.

Finally, the identification of policy effects for almost all policies comes from states implementing the policy in question. However, some states also repealed policies. For this effect estimation, we assume that the effects of implementation and repeal are symmetrical in shape and magnitude, but in opposite directions. Thus, effect estimates are identified by changes in mortality rates after implementation as well as after repeal.

Effect Estimation and Presentation. The primary purpose of the research is to characterize the range of causal effects that are consistent with the available data given this model. Thus, the study uses Bayesian statistical methods, and we describe the posterior distributions of the effects rather than assess their statistical significance. We summarize the posteriors in four ways: (1) the probability that the law or combination of laws reduces mortality of a given type, (2) the posterior median of the total policy effect five years post-implementation, (3) credible intervals corresponding to the central 80% and central 95% regions of the posterior distribution, and (4) figures representing the modelled effect across 6 years following implementation, including the median effect, 80% credible intervals, and a sample from the posterior distribution.

Supplementary Results

Detailed model results. The models of total firearm deaths, firearm suicide, and firearm homicide were estimated with 20,000 MCMC samples. The critical beta parameters all had more than 4,500 effective samples, and all parameters showed Rhat values between 0.999 and 1.001. eTable 3 presents the posterior distribution for key parameters from the model of total firearm deaths. These models have 101 nominal parameters, however, because many of these are subject to hierarchical priors or use somewhat informative priors, the number of effective parameters (Gelman, Hwang, and Vehtari, 2014) is lower. The effective number of parameters were 80, 86 and 77 for total firearm deaths, firearm suicide, and firearm homicide models, respectively. These models are each estimated on 1,950 data points, suggesting there are unlikely to be overfitting problems given the model complexity.

Overall predictive accuracy for these models was excellent. The squared correlations between the posterior median predicted death rate and the observed death rate across all data points were, $R^2 = 0.95$, 0.93, and 0.95, for total firearm deaths, firearm suicide, and firearm homicide outcomes, respectively. The unusually small level of residual variance limits the magnitude of possible bias due to omitted variables.

Effects of individual laws within a law class. The primary results are focused on the effects of six classes of laws, however, four of these classes are subdivided within the model into two nested levels of firearm restrictions that are each allowed to have effects of different sizes. For example, the effect of concealed carry laws is based on the combined effect of two subclasses of laws (1) one law effect represents going from law enforcement discretion in who is allowed to carry a concealed weapon, to *Shall Issue* in where there is no such discretion, and (2) a second effect going from no law enforcement discretion to *Permitless Carry* in which any legal gun owner can carry a concealed weapon. In eTable 4 we present effect estimates for these sub-classes of laws that are the levels of the broader classes included in the primary results.

Effects of laws implemented nationally in 1994. In 1994 the federal government enacted several restrictions on firearm purchase and possession via the *Brady Handgun Violence Prevention Act* and the *Violent Crime Control and Law Enforcement Act of 1994*. Three of the individual laws studied were included in these national changes. Background check requirements for dealer sales and bans on the possession of a handgun by individuals less than 18 years old were both made national law in that year. In addition, one-day waiting periods were temporarily made national policy for states that did not have their own instant background check procedures until that provision sunset with the availability of the National Instant Background Check system. Much of the variation in these three laws occurs in or near 1994, and so it makes sense to look at their combined effect.

However, the model identifies causal effects solely through state-level variation in laws; changes in federal laws that affect all states equally will be fit as part of the year fixed effects. Because of this, the effects we report do not evaluate these federal laws. Rather, the estimated effects are evaluating those state policies that had been implemented prior to 1994, with the national laws playing a role in the identification

© 2024 Schell TL et al. *JAMA Network Open.*

of the causal effect only because the federal laws eliminated heterogeneity across states that previously existed. For example, if the state laws mandating dealer background checks were effective at reducing firearm deaths, then following the federal requirement of background checks gun deaths in the states that did not previously have such policies should improve over time relative to the states that previously did have such policies. The effect size is based on the differences between states that did and did not have background checks prior to 1994 that were eliminated when all states operated under a consistent background check policy. Effects presented in eTable 5 compare the combination of having dealer background checks, one-day waiting periods, and prohibitions on handgun possession for those under 18 to a condition without those three policies. There is some evidence that this combination of state laws was associated with a subsequent reduction in firearm suicides, but little evidence for effects on firearm homicides. However, all of the estimates are relatively uncertain, with the credible intervals covering a descriptively large range of possible effects.

It is plausible that the effects of the national laws are substantially different than the effects of similar policies implemented by individual states. However, because the 5-year national effect of the federal 1994 laws are fit as part of the year effects for 1995-2000 included in the model, we cannot provide strong causal evidence for their effects. Examining those year effect estimates, the trend indicate there was a 33 percent decline in the national firearm mortality rate over that period (IRR=0.67), relative to the model expectation. Of course, many policies, events or conditions occurred in the United States during those years and are also captured in the model's year effects, and so we cannot interpret this 33% decline nationally as the causal effect of the federal firearm laws.

Sensitivity of findings to the chosen priors. Our informative priors for the effects of laws were specified in our preregistration. The effect sizes implied by those priors were taken from a study in which gun policy experts were asked to estimate effect sizes for a wide range of policies and outcomes (Smart, Morral, and Schell, 2021). We replicated the primary results presented in Table 1 and Table 2 using looser priors to address any concerns that the conclusions of the paper depend on the choice of priors (eTable 6). Specifically, we estimated the model with all priors one order of magnitude more dispersed than was used in the primary specification, e.g., a N(0,0.1) prior was replaced with a N(0,1) for this sensitivity test.

The results of this sensitivity test show that, as expected, effect sizes are slightly larger with less informative priors. On average the IRRs with looser priors are 0.005 further from 1 for the total firearm death rate outcome, and their 80% credible intervals are 0.013 wider. These two effects largely compensate for each other when estimating the probability that the laws are beneficial, resulting in little change across the two sets of priors. The use of informative priors makes the largest difference when estimating effects on firearm homicides, which is the sparsest outcome and which has effect estimates with the widest credible intervals. For that outcome, the IRRs with looser priors are 0.027 further from 1 on average, and their 80% credible intervals are 0.042 wider on average. However, these small changes from loosening priors do not affect any of the study conclusions.

Finally, we evaluated whether there was any empirical evidence that the priors were well chosen. Specifically, we compared the leave-one-out cross validated (LOO CV) prediction of the model with our specific, informative priors to one with uninformative priors. For all three outcomes, LOO CV error was lower with the specific, informative priors. This provides some empirical evidence that the priors used in the primary model specification are appropriate for the data.

Sensitivity to inclusion of the *Policy Lagged* (*PL*) spline. One non-standard feature of the primary model was to condition estimates on a "distractor" indicator of treatment, labeled *PL* in equation 1. This is a lagged treatment indicator that begins five years after implementation and phases in linearly over an additional five years. Although this term is derived from the laws, it is not used in our computation of policy effects. We had two reasons for including this term. First, we wished to ensure that our estimates of effects at five years post-implementation were not influenced by variation that occurred substantially later than that—variation that may occur due to other policy or environmental factors that are associated with implementing these laws. Second, the model includes several covariates that are plausible colliders because of likely dependence on either the policies being evaluated or the outcomes. We mitigated that potential problem by lagging those indicators by five years, but this alone would be insufficient to prevent biased estimates beyond five years post-implementation if we allowed effects this late to contribute to our law effect estimates. The *PL* spline is designed to prevent or diminish such biases.

Despite that rationale, the particular covariates in question were weakly associated with these outcomes while controlling for the prior year's outcome, such that the size of any collider bias would be negligible. In addition, this is a type of dynamic time series model which is inherently less sensitive to changes in the outcome that occur many years after implementation. In light of this, it is plausible that effects would be better estimated had we not included those ten *PL* parameters in the model. Including an unnecessary covariate that is associated with the policy indicator could add noise to the policy effect estimates that is larger than the benefits in bias reduction. To address this possibility, we re-ran the models without those terms. For each of the three outcomes, the LOO CV error was slightly better in the simpler model that excluded the *PL* terms. While there are likely some models and datasets for which this approach might be useful for reducing bias in the estimates, it appears unlikely that the potential improvement in estimates is worth the tradeoff in model complexity for these particular data. The policy effects from the simplified model are presented in eTable 7. These estimates are very close to those from our preregistered main model. Across the three outcomes, the average absolute difference in the posterior median IRR between the models with and without the *PL* terms is 0.010. The 95% credible are also slightly narrower by 0.006 on average in the models without *PL* terms. However, none of the estimates changed in a way that would modify our conclusions. Future research may wish to find a more parsimonious approach to ensuring that causal effects are indicated by changes that occur close in time to the hypothesized causes.

Effects of laws on total homicides and suicides. Our primary results focus on firearm deaths because each of these laws was specifically designed to affect firearm-related behaviors, and in most cases, was specifically intended to reduce firearm deaths. Looking at broader classes of deaths that include those which are not directly affected by the policies, i.e., suicides and homicides regardless of lethal means used, is likely to both reduce the effect sizes while also increasing the variance of the estimates. These statistical effects make it harder to identify which laws are beneficial versus harmful when looking at estimated effects on total suicide and homicide rates.

Having said that, some have theorized that gun policies may have important second-order effects on non-firearm homicides and suicides (see Smart, et al., 2020 and Braga et al., 2021 for reviews). For example, suicides by hanging might increase in response to increased difficulty in buying a firearm, or policies that increase firearm homicides might promote retaliatory violence that includes both firearm and non- firearm homicide. To determine whether such second-order effects meaningfully modify the study conclusions we estimate our primary model on total suicides and total homicides (regardless of means). These estimates are presented in eTable 8. The effects show a general attenuation of the effects estimated on firearm homicide and firearm suicide. The combined effects of all policies restricting firearms, labeled *Most Restrictive*, were IRR= 0.92 on total suicide compared with our estimate of IRR=0.81 for firearm suicide outcomes. Thus, the effect size decreases by about half when including the non-firearm suicides, a decline in size that is broadly similar to the proportion of suicides that did not use a firearm (0.45) over the study period. Similarly, the combined effects of all policies were IRR= 0.91 versus IRR=0.81 on total homicide and firearm homicide outcomes, respectively. For homicides, the effect size decreases by slightly less than half when including non-firearm homicides in the outcome. This decline is also broadly consistent with, although somewhat larger than, the proportion of homicides that did not use a firearm (0.33). These results are consistent with the view that the effects of firearm laws on mortality are primarily driven by changes in deaths caused by firearms, with generally small second-order effects on non-firearm mortality.

Causal effect estimates using alternative statistical methods. Readers may be interested in comparing effects of the primary model to those from alternative statistical methods for estimating causal effects. In this section we estimate effects using the method described in Callaway and Sant'Anna (2021) which can be seen as a stacked-trial emulation approach to policy evaluation (Ben-Michael, Feller, and Stuart, 2021). Below, we describe the specific methods used in implementing this approach, compare the effect estimates to those from our primary specification, and describe the limitations of this approach for the purposes of the current study.

The effect estimates are based on the staggered adoption difference-in-differences method of Callaway and Sant'Anna (2021), and use a linear model of state firearm mortality rates weighted by state population. This approach produces a separate estimate of the effect of each policy for each "cohort" (where cohorts are defined by the year of policy implementation) in each post-treatment year by comparing the difference between treatment and controls in that year to the difference between treatment and controls in the last pre-treatment year. The control states in a given year include the never-treated states as well as

© 2024 Schell TL et al. *JAMA Network Open.*

the not-yet-treated states that are available in that post-treatment year for each treated cohort. Overall effects can then be produced by averaging effects over the cohorts and over the post-period . Finally, we convert effects expressed as differences in rates to rate ratios to make them more comparable to the effects from the primary model.

This effect estimation method requires a dichotomous treatment transition from no treatment to treatment for each treated unit in the analysis, meaning units that are treated over the entire study period are excluded from all analyses. Unlike the primary model it will not accept treatments for partial time periods, it does not accommodate multiple different treatments, nor can it use information from changes in mortality that follow policy repeals in estimating treatment effects. For these reasons we estimate separate treatment effects for each of the ten underlying dichotomous policies included in the study. This is done by counting the first year with the policy in force for more than six months as being treated beginning in that year, so long as the policy was not repealed in the subsequent five years. Under this coding, policies that are in effect for less than five years (less than half the post-period) are effectively treated as control units in the analysis, while repeals of policies that were in force for more than five years are ignored and the state is coded as continuously treated after initial implementation. Because of these restrictions on how treatments can be defined and how the comparison groups are constructed, the effect estimates using this method rely on fewer policy transitions to identify the policy effects. eTable 9 contains information about the number of transitions that were ultimately used in each estimate.

The pattern of these effect estimates (eTable 9) is descriptively similar to the primary model. Each of the seven policies that represent a firearm restriction was associated with a small reduction in deaths (IRR < 1) and each of the three permissive policies was associated with a small increase in deaths (IRR > 1). The effects of these policies evaluated individually, however, were not statistically significant at the $p < 0.05$ level. The effect estimates were, on average, slightly larger in magnitude than our primary results. For example, if you were to treat these trial emulation estimates of individual policies as independent estimates with additive effects, the total effect of transitioning from the lowest restrictiveness policy regime to the highest corresponds to an IRR = 0.76, a 24% reduction in firearm mortality.

However, these estimates are not designed to be combined in this manner, and those combined estimates may be substantially inaccurate. Unlike the primary model, these estimates do not take into account the other (often highly correlated) policies being studied. For example, a state that implements a particular firearm restriction, such as CAP is also highly likely to implement additional restrictive policies in the next several years. Any decrease in mortality observed beyond that point is "double-counted" as an effect of both CAP and as effect of the subsequent restrictive policy. This may bias these estimates to overestimate the effects firearm restrictions. In contrast the primary model simultaneously estimates the effects of all policies, accounting for the joint effects of correlated or co-occurring policies.

Although the staggered adoption DiD approach of Callaway and Sant'Anna (2021) does not allow controlling for post-treatment factors, such as other policies, it does allow for balancing treatment and controls on pre-treatment characteristics. In practice, however we were not able get estimates from the models when including multiple pre-treatment covariates. Because of that, the estimates we provide above have not been balanced the treatment and controls on pre-treatment covariates.

Because the stacked DiD methods require dichotomous treatment indictors we rounded our fractional treatment indicators for the implementing year. This results in effects where the "pre" treatment year often includes a partial treatment effect (up to six months), and the first post-treatment year is often only partly exposed to treatment. These approximations necessarily result in a bias toward underestimating the true magnitude of causal effect.

Finally, the Callaway and Sant'Anna method computes causal effects under a parallel trends assumption. For each policy, we conducted tests for deviations in trend between treated and control states based on the ten years preceding policy adoption. For all policies, these tests rejected the null that all pre-treatment differences were statistically equal to zero, suggesting statistically significant deviations from parallel trends for all analyses.

Supplementary Discussion

Discussion of the homogeneous effects assumption. One of the key assumptions for these estimates is that the effects of all policies are well approximated as a constant risk ratio, i.e., a policy causes firearm mortality to be a constant percentage higher or lower than would be expected without the policy. This is built into the model because it assumes effects are additive on a log-rate scale. Thus, we assume the risk ratio for a given law does not change based on what other laws are present, what year it was implemented,

or the values of the covariates. To clarify, this is not the same as assuming the policies have a constant effect expressed as a change in the mortality rate – a much more common assumption in the existing literature. That assumption appears to be implausible over the range of mortality rates across states. For example, it may be reasonable to believe a set of firearm polices would reduce Wyoming's annual firearm mortality by 4 — representing a shift from approximately 26 to 22 deaths per 100,000, a reduction of 15% — but Hawaii has a firearm mortality rate of only 3.6 per 100,000 and it is impossible to reduce it by 4 regardless of how effective the policies might be. Our assumption of a constant rate ratio implies a particular interaction on a rate scale such that all effects of policies result in larger changes in the firearm mortality rate for years and states with higher firearm mortality rates than for years and states with lower mortality rates. In this section we discuss the possible model misspecification due to this homogeneous effect assumption, including bounding how large the misspecification could be, presenting a joint test of laws that may be more robust to this misspecification, and a discussion of challenges for any efforts to relax these assumptions.

The possible model misspecification caused by assuming the policies have constant rate ratios is a subtype of a broader form of model misspecification due to omitted variables, in this case omitted interactions involving policies. The magnitude of that larger set of possible misspecifications is bounded by the variance of the model residual. The models we interpret explain 95% of the variance in the observed state level firearm mortality over time, leaving 5% of that variance as residual. In spite of the relatively restrictive additivity assumption, we know that the total variance potentially explainable by all possible omitted variables and interactions is a very small fraction of the variance explained by the existing model. Globally, there is strong reason to believe that the simplifying assumption of additivity is fully appropriate for the most important relationships in these data.

However, it is possible that omitted interactions are meaningfully large relative to the small effects for firearm policies we are estimating in this model. To the extent that this constant IRR assumption is incorrect our results are more robust if we are using the model to interpolate within the data used to estimate the model rather than extrapolate beyond it. If we are estimating effects within the range of values that are well covered by the data, the estimates are likely good approximations because the estimates are well constrained by the data. If we are using the model assumption to extrapolate outside the data on which the model was estimated, the expected values may be relatively inaccurate when the model assumptions are violated. In general, the seven restrictive firearm policies are strongly positively correlated with each other and negatively correlated with the three permissive policies. Because of this, the particular combinations of policies we jointly test are located near the densest portions of the multivariate policy space. With two minor exceptions where federal law has superseded state law, all law combinations we test occur within the data. Looking at the eight policies where federal law currently allows variation in the most recent year of the data, 10 states had the most permissive set (AK, AZ, ID, KS, KY, MO, MS, OK, SD, WV) and 5 states (CA, HI, MD, NJ, RI) had the most restrictive set. For those 8 policies, thirty percent of the states are found in the two extreme position within the policy space, and those combinations are the modes of the joint policy distribution. Most of the remaining states that are not exactly at those modes are clustered within two policy transitions from those locations; sixty percent of all states are within a single policy transition from being in the most restrictive or most permissive policy combination. The middle of the multivariate policy space is essentially empty, with only one state currently having an even mix of permissive and restrictive policies.

This analysis ignores the two policies where permissive state policies have been superseded by federal restrictions: background checks for FFL dealer sales and minimum age of handgun possession laws. The states with otherwise permissive policies are constrained by federal laws passed in 1994. Because of that, some combinations of permissive policies involving these laws do not occur in the data, and others occur rarely. To address concerns that our overall comparison of permissive to restrictive policy regimes require extrapolations to locations that are outside the estimating data, we include a sensitivity test that excludes these two policies from our joint test comparison. This effectively assesses permissive verses restrictive policy combinations at the locations in the joint policy space that are currently the empirical modes. This joint estimate of restrictive to permissive policies is IRR= 0.86 (80% CI 0.77-0.95) for total firearm deaths.

It may be possible to relax the constant IRR assumption for some policies, and the field would benefit from further research into the plausibility of these assumptions. However, any effort to explore those interactions empirically will need to work within the limits of the data. There are far more possible interactions involving the policy variables than can be estimated in the data. Among the 10 policy variables

© 2024 Schell TL et al. *JAMA Network Open.*

themselves there are 1014 possible interactions, with a much broader set if one includes interactions with other important model variables such as potential confounds, time, or the prior year's mortality rate. Given there are only a couple hundred policy transitions across all of the studied policies, specifying a set of interactions in such a model would need to be a narrow, theory-guided decision, and may still require substantial statistical regularization.

Discussion of the criteria for a target trial emulation. The current study has the components of a target trial emulation (Hernán, 2020), which have been proposed as desirable features for studies attempting to infer causation from observational data . In the section below, we address each component:

Protocol Component:	Response:
Eligibility criteria	All residents of the 50 United States are included in the trial. (Non-US residents, those from the District of Columbia, and US territories are excluded)
Treatment strategies	All treatments being investigated are implemented at the state level. The precise definition of the treatments, and the data used to code assignment to treatments were publicly published and pre-registered prior to the analysis.
Treatment assignment	Treatments were determined by state statutes (or federal supersession of state statutes) which applied to all individuals residing in the affected state
Outcomes	Risk for specific forms of mortality. Forms were specified in our pre-registration and mortality was assessed by the national death records in the National Vital Statistics System. The sizes of the state resident population in each year used to convert mortality counts to rates were taken from National Center for Health Statistics, which at the state level are almost identical to the Census Bureau's final intercensal population estimates for these years.
Follow-up	Outcomes were assessed 1/1/1981 through 12/31/2019.
Causal estimand	Per Protocol. Residents within a state are analyzed as "treated" regardless of whether the officials in the state implemented the policies specified in the statues in a timely or faithful manner. Individuals contribute to the firearm death rate of their state of residence even if the death occurred while out of state (e.g., in an untreated state).
Statistical analysis	Effects are estimated in a longitudinal model of change in mortality risk, estimated at the state level. Extensive prior statistical simulations demonstrate the method accurately recovers true causal effects that were added to state-level firearm mortality data better than more than 100 candidate statistical methods tested (Schell, Griffin & Morral , 2018, Cefalu et al, 2021). The model includes 31 covariates, including several that have been hypothesized as possible serious confounds. In addition, the various firearm policies studied all correlate with one another and represent potential confounds. The joint estimation method ensures that the effect of each policy reflects its association with other policies in both the estimate itself and the stated uncertainty in the estimate. The researchers are not aware of specific constructs proposed as confounds within the literature that we have not attempted to control for with one or more included covariates. The model, outcomes, covariates, priors, and effects to be estimated were all specified in a pre-registration before estimating any effects.

Discussion of the relationship to Schell et al, 2020b. The current study has some overlap in both data and methods with the authors' prior published work. The current study substantially extends the earlier study. To the extent that some estimates conceptually overlap between them, the current study should be seen as superseding those earlier estimates. The current study: (a) estimates the joint effect of 10 policies, rather than 3, (b) has better protection against confounding due to the inclusion of controls for gun ownership rates and political control of the states, (c) uses a model that better fits the data, (d) requires the changes in

mortality rates that identify the effects of policies to follow more closely in time to the implementation of the polices, and (e) adds additional years of data.

© 2024 Schell TL et al. *JAMA Network Open.*

eTable 1. Years of Implementation for Firearms Policies

eTable 1a. Years of implementation for laws governing purchase and possession of firearms over the studied period by state.

State	Dealer Background Check	Universal Background Check	Waiting Period 1 day	Waiting Period 7 days	Minimum age 18+ possess	Minimum age 20+ purchase
Alabama	1994–2020	NA	1975–2000	NA	1994–2020	NA
Alaska	1994–2020	NA	1994–1998	NA	1994–2020	NA
Arizona	1994–2020	NA	1994–1994	NA	1993–2020	NA
Arkansas	1994–2020*	NA	1994–1997	NA	1989–2020	NA
California	1975–2020	1991–2020	1975–2020	1976–2020	1975–2020	1990–2020
Colorado	1994–2020	2013–2020	NA	NA	1993–2020	NA
Connecticut	1994–2020	1994–2020	1975–1995	1975–1995	1994–2020	1994–2020
Delaware	1990–2020	1994–2020	NA	NA	1994–2020	1987–2020
Florida	1990–2020	NA	1991–2020	NA	1994–2020	2018–2020
Georgia	1994–2020	NA	1994–1996	NA	1994–2020	1975–1994
Hawaii	1975–2020	1975–2020	1988–2020	1988–2020	1994–2020	1994–2020
Idaho	1994–2020	NA	1994–1994	NA	1994–2020	NA
Illinois	1975–2020	1975–2020	1975–2020	NA	1975–2020	1975–2020
Indiana	1983–2020	1983–1998	1975–1998	1975–1998	1994–2020	1975–1977
Iowa	1990–2020	1990–2020	1978–2020	1978–2020	1979–2020	1979–2020
Kansas	1994–2020	NA	1994–1998	NA	1994–2020	NA
Kentucky	1994–2020	NA	1994–1998	NA	1994–2020	NA
Louisiana	1994–2020	NA	1994–1998	NA	1994–2020	NA
Maine	1994–2020	NA	1994–1998	NA	1994–2020	NA
Maryland	1975–2020	1996–2020	1975–2020	1975–2020	1994–2020	1975–2020
Massachusetts	1975–2020	1975–2020	NA	NA	1994–2020	1998–2020
Michigan	1975–2020	NA	NA	NA	1991–2020	NA
Minnesota	1977–2020	NA	1977–2020	1977–2020	1975–2020	NA
Mississippi	1994–2020	NA	1994–1998	NA	1994–2020	NA
Missouri	1975–2020	1975–2007	NA	NA	1994–2020	1981–2007
Montana	1994–2020	NA	1994–1998	NA	1994–2020	NA
Nebraska	1991–2020	1991–2020	1991–2020	1991–2020	1977–2020	1991–1994
Nevada	1994–2020	1997–2020	NA	NA	1994–2020	NA
New Hampshire	1994–2020	NA	1994–1995	NA	1994–2020	NA
New Jersey	1975–2020	1975–2020	1979–2020	1979–2020	1975–2020	2001–2020
New Mexico	1994–2020	2019–2020	1994–1998	NA	1994–2020	NA
New York	1975–2020	1975–2020	NA	NA	1994–2020	2000–2020
North Carolina	1975–2020	1975–2020	1975–2020	1975–2020	1993–2020	NA
North Dakota	1994–2020	NA	1994–1998	NA	1985–2020	NA
Ohio	1994–2020*	NA	NA	NA	1994–2020	1975–2020
Oklahoma	1994–2020	NA	1994–1998	NA	1993–2020	NA
Oregon	1990–2020	2015–2020	1975–1996	1990–1996	1990–2020	NA
Pennsylvania	1994–2020	1998–2020	1975–1998	NA	1994–2020	NA
Rhode Island	1975–2020	1975–2020	1975–2020	1990–2020	1994–2020	1975–2020
South Carolina	1994–2020	NA	NA	NA	1975–2020	1975–1998
South Dakota	1994–2020	NA	1975–2009	NA	1994–2020	NA
Tennessee	1975–2020	1975–1998	1975–1998	1989–1998	1994–2020	NA
Texas	1994–2020	NA	1994–1998	NA	1994–2020	NA
Utah	1994–2020	NA	NA	NA	1993–2020	NA
Vermont	1994–2020	2018–2020	1994–1998	NA	1994–2020	2018–2020
Virginia	1989–2020	NA	NA	NA	1993–2020	NA
Washington	1975–2020	2014–2020	1975–2020	2014–2020	1994–2020	1975–1994; 2019–2020
West Virginia	1994–2020	NA	1994–1998	NA	1989–2020	NA
Wisconsin	1991–2020	NA	1976–2015	NA	1975–2020	NA
Wyoming	1994–2020	NA	1994–1998	NA	1994–2020	2010–2020

Note: NA indicates the specific policy was never implemented during the study period. Study period covers January 1975 to January 2020. Laws implemented prior to 1975 are listed as starting in 1975, the beginning of the study period. *Arkansas and Ohio had temporary discontinuations of requirements for dealers to conduct background checks related to *Printz v. United States*. We treat these states as not having federal dealer background check requirements for partial periods in 1997 and 1998. Detailed information about effective dates and law citations are provided in the RAND State Firearm Law Database.

© 2024 Schell TL et al. *JAMA Network Open.*

eTable 1b. Years of implementation for laws governing firearm storage and use over the studied period by state.

State	Child Access Prevention	Stand your ground*	Shall Issue*	Permitless Carry*
Alabama	NA	2006–2020	1975–2020	NA
Alaska	NA	2013–2020	1994–2020	2003–2020
Arizona	NA	2006–2020	1994–2020	2010–2020
Arkansas	NA	NA	1995–2020	2018–2020
California	1992–2020	NA	NA	NA
Colorado	NA	NA	2003–2020	NA
Connecticut	1990–2020	NA	1975–2020	NA
Delaware	1994–2020	NA	NA	NA
Florida	1989–2020	2005–2020	1987–2020	NA
Georgia	NA	2006–2020	1989–2020	NA
Hawaii	1992–2020	NA	NA	NA
Idaho	NA	2018–2020	1990–2020	2016–2020
Illinois	2000–2020	NA	2013–2020	NA
Indiana	NA	2006–2020	1980–2020	NA
Iowa	1990–2020	2017–2020	2011–2020	NA
Kansas	NA	2006–2020	2007–2020	2015–2020
Kentucky	NA	2006–2020	1996–2020	2019–2020
Louisiana	NA	2006–2020	1996–2020	NA
Maine	NA	NA	1985–2020	2015–2020
Maryland	1992–2020	NA	NA	NA
Massachusetts	1998–2020	NA	NA	NA
Michigan	NA	2006–2020	2001–2020	NA
Minnesota	1993–2020	NA	2003–2020	NA
Mississippi	NA	2006–2020	1990–2020	2016–2020
Missouri	NA	2016–2020	2004–2020	2017–2020
Montana	NA	2009–2020	1991–2020	NA
Nebraska	NA	NA	2007–2020	NA
Nevada	1991–2020	2011–2020	1995–2020	NA
New Hampshire	2001–2020	2011–2020	1975–2020	2017–2020
New Jersey	1992–2020	NA	NA	NA
New Mexico	NA	NA	2004–2020	NA
New York	2019–2020	NA	NA	NA
North Carolina	1993–2020	2011–2020	1995–2020	NA
North Dakota	NA	NA	1985–2020	2017–2020
Ohio	NA	2019–2020	2004–2020	NA
Oklahoma	NA	2006–2020	1996–2020	2019–2020
Oregon	NA	NA	1990–2020	NA
Pennsylvania	NA	2011–2020	1989–2020	NA
Rhode Island	1995–2020	NA	NA	NA
South Carolina	NA	2006–2020	1996–2020	NA
South Dakota	NA	2006–2020	1985–2020	2019–2020
Tennessee	NA	2007–2020	1996–2020	NA
Texas	1995–2020	2007–2020	1996–2020	NA
Utah	NA	1994–2020	1995–2020	NA
Vermont	NA	NA	1975–2020	1975–2020
Virginia	1991–2020	NA	1995–2020	NA
Washington	2019–2020	NA	1975–2020	NA
West Virginia	NA	2008–2020	1989–2020	2016–2020
Wisconsin	1992–2020	NA	2011–2020	NA
Wyoming	NA	2018–2020	1994–2020	2011–2020

Note: NA indicates the specific policy was never implemented during the study period. Study period covers January 1975 to January 2020. Detailed information about effective dates and law citations are provided in the RAND State Firearm Law Database. * Indicates policies that create a more permissive firearm policy environment when implemented; all others create a more restrictive environment.

© 2024 Schell TL et al. *JAMA Network Open.*

eTable 2. State Characteristics Included as Covariates

Type	Variable	Notes
Possible Confounds		
	Household gun ownership rate	*
	Political control of state	*
	UCR Robbery + Agg Assault Crime Rate	*
	Incarcerated persons per capita	*
	Proportion of income for top 10%	
Other State Characteristics		
	Percent unemployed (U3)	
	Change in unemployment from prior	
	Average income (inflation adjusted)	
	Poverty rate	
	Percent younger than 15	
	Percent 15–29	
	Percent 30–59	†
	Percent 60 and over	
	Percent Caucasian	†
	Percent African American	
	Percent Asian/Pacific Islander	
	Percent American Indian/Alaska Native	
	Percent Hispanic	
	Percent Married	†
	Percent Divorced/Separated/Widowed	
	Percent Never married	
	Percent bachelors or higher	
	Gender ratio	
	Percent children single parent household	
	Percent foreign born	
	Percent military veterans	
	Percent urban households	
	Percent >25 years old, Black, and urban	
	Wave of U.S. expansion	
	Population Density	
	Alcohol consumption per capita	

Note: * indicates variables that were judged to be possible colliders, i.e., they may be affected by the firearm laws or by firearm deaths. To minimize biases caused by this, they were lagged by 5 years, e.g., the 1995 version of the covariate is used when predicting outcomes in 2000. † indicates variables that are treated as referents; they are included in the model by virtue of being linearly dependent on the other variables included in the model.

© 2024 Schell TL et al. *JAMA Network Open.*

eTable 3. Posterior Medians and 95% Credible Intervals for Model Parameters by Type of Firearm Death

	Total Firearm Deaths		Firearm Suicides		Firearm Homicides	
**Parameter**	**Median**	**95% CI**	**Median**	**95% CI**	**Median**	**95% CI**
alpha	-0.60	(-0.807, -0.414)	-1.11	(-1.396, -0.835)	-0.83	(-1.050, -0.609)
delta1	0.59	(0.551, 0.632)	0.46	(0.424, 0.502)	0.55	(0.512, 0.588)
delta2	0.34	(0.301, 0.382)	0.42	(0.380, 0.457)	0.37	(0.331, 0.407)
PI.CAP	-0.01	(-0.047, 0.025)	0.01	(-0.025, 0.048)	-0.05	(-0.107, 0.013)
PI.SYG	0.03	(0.001, 0.066)	0.01	(-0.023, 0.041)	0.07	(0.012, 0.125)
PI.SI	0.03	(-0.002, 0.053)	0.02	(-0.010, 0.045)	0.07	(0.023, 0.119)
PI.PC	0.02	(-0.025, 0.069)	0.02	(-0.029, 0.065)	0.03	(-0.044, 0.111)
PI.UBC	0.03	(-0.008, 0.073)	0.02	(-0.023, 0.058)	0.06	(-0.010, 0.123)
PI.DBC	-0.04	(-0.072, -0.005)	-0.04	(-0.075, -0.008)	-0.04	(-0.099, 0.015)
PI.MA18	-0.03	(-0.070, 0.001)	-0.02	(-0.058, 0.014)	-0.04	(-0.096, 0.023)
PI.MA20	0.00	(-0.037, 0.040)	0.00	(-0.037, 0.040)	-0.01	(-0.069, 0.057)
PI.WP1	0.00	(-0.029, 0.032)	0.01	(-0.024, 0.038)	0.01	(-0.040, 0.066)
PI.WP7	-0.05	(-0.100, 0.011)	-0.03	(-0.083, 0.025)	-0.03	(-0.117, 0.053)
PS.CAP	-0.05	(-0.110, 0.009)	-0.04	(-0.091, 0.020)	-0.04	(-0.131, 0.045)
PS.SYG	0.00	(-0.058, 0.050)	0.01	(-0.040, 0.060)	-0.01	(-0.091, 0.077)
PS.SI	0.00	(-0.042, 0.048)	0.01	(-0.031, 0.055)	-0.02	(-0.092, 0.054)
PS.PC	0.00	(-0.088, 0.081)	-0.01	(-0.087, 0.070)	-0.01	(-0.121, 0.106)
PS.UBC	-0.01	(-0.066, 0.058)	-0.01	(-0.068, 0.046)	0.01	(-0.085, 0.101)
PS.DBC	0.03	(-0.027, 0.092)	-0.01	(-0.063, 0.051)	0.04	(-0.047, 0.135)
PS.MA18	-0.02	(-0.085, 0.040)	0.00	(-0.064, 0.056)	-0.03	(-0.125, 0.062)
PS.MA20	0.00	(-0.067, 0.066)	-0.04	(-0.102, 0.020)	0.03	(-0.062, 0.128)
PS.WP1	0.01	(-0.049, 0.061)	-0.02	(-0.074, 0.031)	0.03	(-0.052, 0.115)
PS.WP7	-0.01	(-0.084, 0.068)	0.02	(-0.049, 0.092)	-0.02	(-0.122, 0.088)
PL.CAP	0.00	(-0.056, 0.059)	-0.01	(-0.068, 0.040)	0.01	(-0.073, 0.100)
PL.SYG	0.02	(-0.040, 0.072)	0.01	(-0.047, 0.057)	0.03	(-0.061, 0.113)
PL.SI	0.01	(-0.032, 0.055)	0.03	(-0.011, 0.072)	-0.02	(-0.089, 0.054)
PL.PC	0.02	(-0.089, 0.128)	0.03	(-0.067, 0.119)	0.00	(-0.122, 0.129)
PL.UBC	-0.02	(-0.056, 0.014)	-0.01	(-0.047, 0.025)	-0.06	(-0.114, 0.003)
PL.DBC	0.04	(-0.015, 0.101)	0.03	(-0.023, 0.085)	0.03	(-0.062, 0.115)
PL.MA18	-0.02	(-0.081, 0.038)	0.00	(-0.054, 0.056)	-0.04	(-0.133, 0.047)
PL.MA20	0.00	(-0.030, 0.024)	0.01	(-0.022, 0.035)	0.00	(-0.045, 0.048)
PL.WP1	0.01	(-0.012, 0.033)	0.01	(-0.013, 0.035)	0.01	(-0.027, 0.053)
PL.WP7	0.02	(-0.023, 0.064)	0.00	(-0.047, 0.042)	0.05	(-0.024, 0.120)
Hshold.Firearm.Rate	0.02	(0.007, 0.027)	0.03	(0.014, 0.040)	0.01	(-0.004, 0.028)
GOP.control	0.00	(-0.004, 0.004)	0.00	(-0.003, 0.006)	0.00	(-0.010, 0.005)
Incarceration.Rate	0.00	(-0.002, 0.008)	0.00	(-0.005, 0.006)	0.01	(0.001, 0.020)
Robbery.Assault.Rate	0.00	(-0.002, 0.009)	0.00	(-0.004, 0.007)	0.01	(0.001, 0.022)
Decile.income.share	0.00	(-0.009, 0.001)	0.00	(-0.007, 0.005)	-0.01	(-0.018, 0.001)
Unemployment.Rate	0.00	(-0.004, 0.002)	0.00	(-0.005, 0.005)	0.00	(-0.009, 0.005)
Income2000dollars	0.00	(-0.006, 0.002)	-0.01	(-0.015, 0.001)	0.00	(-0.008, 0.007)
Poverty.Rate	0.00	(-0.003, 0.004)	0.00	(-0.004, 0.006)	0.00	(-0.007, 0.006)
Unemployment.change	0.00	(-0.002, 0.006)	0.00	(-0.001, 0.009)	0.00	(-0.002, 0.014)
Alcohol.per.capita	0.00	(-0.002, 0.004)	0.00	(-0.003, 0.006)	0.00	(-0.005, 0.007)
Gender.Ratio	0.00	(-0.005, 0.004)	0.01	(-0.002, 0.019)	0.00	(-0.013, 0.005)
Percent.0.to.14	0.00	(-0.004, 0.003)	0.00	(-0.008, 0.004)	0.00	(-0.008, 0.007)
Percent.15.to.29	0.00	(-0.002, 0.005)	0.00	(-0.002, 0.007)	0.00	(-0.006, 0.007)
Percent.60.and.over	0.00	(-0.003, 0.003)	0.00	(-0.006, 0.005)	0.00	(-0.006, 0.008)
Percent.Never.Married	0.00	(-0.004, 0.003)	0.00	(-0.009, 0.003)	0.00	(-0.005, 0.009)
Percent.Div.Sep.Wid	0.00	(-0.002, 0.006)	0.00	(-0.002, 0.010)	0.00	(-0.004, 0.011)
Percent.Child.1.Parent	0.00	(-0.002, 0.005)	0.00	(-0.006, 0.003)	0.00	(-0.002, 0.014)
Percent.Hispanic	0.00	(-0.002, 0.006)	0.00	(-0.002, 0.010)	0.00	(-0.004, 0.013)
Percent.Black	0.00	(-0.002, 0.008)	0.00	(-0.004, 0.009)	0.01	(-0.001, 0.023)
Percent.AIAN	0.00	(-0.003, 0.003)	0.00	(-0.005, 0.005)	0.00	(-0.007, 0.007)

© 2024 Schell TL et al. *JAMA Network Open.*

	Total Firearm Deaths		Firearm Suicides		Firearm Homicides	
**Parameter**	**Median**	**95% CI**	**Median**	**95% CI**	**Median**	**95% CI**
Percent.API	0.00	(-0.007, 0.002)	-0.01	(-0.017, 0.001)	0.00	(-0.008, 0.008)
Percent.Urban.Hsholds	0.00	(-0.003, 0.005)	0.00	(-0.004, 0.007)	0.00	(-0.004, 0.014)
Percent.Yng.Blck.Urbn	0.00	(-0.002, 0.007)	0.00	(-0.005, 0.008)	0.01	(-0.002, 0.019)
Percent.college.degree	0.00	(-0.003, 0.003)	0.00	(-0.004, 0.006)	0.00	(-0.009, 0.005)
Interp.Foreign.Born	0.00	(-0.007, 0.002)	0.00	(-0.009, 0.005)	0.00	(-0.014, 0.004)
Percent.Veterans	0.00	(0.000, 0.008)	0.01	(0.004, 0.016)	0.00	(-0.005, 0.007)
Population.density	0.00	(-0.006, 0.003)	-0.01	(-0.019, 0.001)	0.00	(-0.007, 0.010)
Frontier.wave	0.00	(-0.001, 0.007)	0.00	(-0.002, 0.009)	0.00	(-0.005, 0.009)
tau	0.00	(0.000, 0.004)	0.00	(0.002, 0.007)	0.00	(0.002, 0.007)
Year 1982	-0.03	(-0.061, -0.006)	0.01	(-0.023, 0.038)	-0.1	(-0.145, -0.046)
Year 1983	-0.07	(-0.098, -0.043)	-0.01	(-0.043, 0.019)	-0.17	(-0.222, -0.123)
Year 1984	-0.02	(-0.047, 0.011)	0.02	(-0.016, 0.049)	-0.07	(-0.121, -0.016)
Year 1985	0.00	(-0.029, 0.026)	0.00	(-0.027, 0.034)	0.00	(-0.046, 0.052)
Year 1986	0.05	(0.024, 0.080)	0.03	(0.003, 0.065)	0.09	(0.044, 0.143)
Year 1987	0.00	(-0.024, 0.031)	0.01	(-0.018, 0.046)	-0.01	(-0.057, 0.042)
Year 1988	0.01	(-0.022, 0.034)	-0.02	(-0.049, 0.014)	0.04	(-0.013, 0.087)
Year 1989	0.02	(-0.005, 0.051)	-0.01	(-0.044, 0.018)	0.08	(0.027, 0.126)
Year 1990	0.06	(0.035, 0.090)	0.03	(-0.003, 0.060)	0.11	(0.060, 0.160)
Year 1991	0.04	(0.009, 0.065)	-0.02	(-0.052, 0.013)	0.12	(0.066, 0.168)
Year 1992	-0.01	(-0.036, 0.020)	-0.06	(-0.088, -0.025)	0.05	(-0.001, 0.098)
Year 1993	0.05	(0.017, 0.073)	0.01	(-0.023, 0.038)	0.09	(0.037, 0.136)
Year 1994	0.03	(-0.008, 0.057)	0.01	(-0.028, 0.042)	0.06	(0.007, 0.119)
Year 1995	-0.04	(-0.075, -0.008)	-0.02	(-0.052, 0.021)	-0.06	(-0.116, 0.000)
Year 1996	-0.07	(-0.101, -0.038)	-0.04	(-0.074, -0.005)	-0.12	(-0.172, -0.063)
Year 1997	-0.08	(-0.108, -0.046)	-0.07	(-0.102, -0.033)	-0.09	(-0.142, -0.035)
Year 1998	-0.08	(-0.116, -0.052)	-0.06	(-0.090, -0.021)	-0.12	(-0.180, -0.071)
Year 1999	-0.09	(-0.119, -0.053)	-0.08	(-0.112, -0.042)	-0.1	(-0.160, -0.049)
Year 2000	-0.05	(-0.086, -0.021)	-0.06	(-0.096, -0.027)	-0.05	(-0.108, 0.002)
Year 2001	0.00	(-0.036, 0.029)	-0.03	(-0.069, 0.002)	0.02	(-0.034, 0.078)
Year 2002	0.01	(-0.028, 0.038)	-0.03	(-0.061, 0.010)	0.03	(-0.024, 0.088)
Year 2003	-0.02	(-0.048, 0.017)	-0.05	(-0.084, -0.014)	0.02	(-0.034, 0.076)
Year 2004	-0.03	(-0.066, -0.001)	-0.05	(-0.085, -0.015)	-0.01	(-0.065, 0.044)
Year 2005	0.01	(-0.020, 0.039)	-0.03	(-0.063, 0.003)	0.06	(0.005, 0.108)
Year 2006	0.00	(-0.033, 0.026)	-0.04	(-0.072, -0.007)	0.05	(-0.003, 0.099)
Year 2007	-0.02	(-0.046, 0.013)	-0.03	(-0.057, 0.007)	-0.02	(-0.069, 0.033)
Year 2008	0.00	(-0.027, 0.031)	0.01	(-0.018, 0.047)	-0.05	(-0.100, 0.004)
Year 2009	-0.03	(-0.057, 0.006)	-0.01	(-0.045, 0.029)	-0.08	(-0.143, -0.027)
Year 2010	0.00	(-0.027, 0.033)	0.01	(-0.021, 0.046)	-0.05	(-0.100, 0.005)
Year 2011	0.01	(-0.022, 0.037)	0.01	(-0.021, 0.045)	-0.02	(-0.076, 0.030)
Year 2012	0.03	(-0.003, 0.055)	0.02	(-0.014, 0.052)	0.03	(-0.021, 0.085)
Year 2013	0.00	(-0.031, 0.028)	0.01	(-0.020, 0.044)	-0.04	(-0.089, 0.015)
Year 2014	-0.02	(-0.046, 0.012)	0.00	(-0.034, 0.031)	-0.05	(-0.100, 0.005)
Year 2015	0.06	(0.029, 0.086)	0.01	(-0.023, 0.041)	0.16	(0.113, 0.216)
Year 2016	0.07	(0.045, 0.102)	0.03	(-0.001, 0.061)	0.16	(0.106, 0.206)
Year 2017	0.04	(0.013, 0.069)	0.04	(0.006, 0.068)	0.04	(-0.007, 0.091)
Year 2018	0.00	(-0.027, 0.029)	0.02	(-0.011, 0.050)	-0.02	(-0.071, 0.027)
Year 2019	-0.01	(-0.034, 0.021)	-0.02	(-0.054, 0.009)	0.03	(-0.024, 0.076)
Inverse phi	0.00	(0.002, 0.003)	0.00	(0.002, 0.002)	0.01	(0.007, 0.010)

Note: Policy and year effects are for variables bounded 0 to 1. Covariate effects are for standardized continuous variables.

© 2024 Schell TL et al. *JAMA Network Open.*

eTable 4. Effect Sizes of Law Subclasses on State Firearm Death Rates in the Fifth Year After Implementation, for Law Classes With Multiple Levels

**Outcome**<br>**Law Class**	**Posterior median IRR**	**80% CI Lower**	**80% CI Upper**	**95% CI Lower**	**95% CI Upper**	**Posterior probability of reduced deaths**
Firearm Deaths						
Background Checks						
Dealer BC	0.99	0.95	1.04	0.93	1.06	0.58
Universal BC	1.03	0.99	1.07	0.96	1.10	0.20
Minimum Age						
Possession 18+	0.95	0.90	0.99	0.88	1.01	0.95
Purchase 20+	1.00	0.96	1.05	0.94	1.07	0.48
Waiting Periods						
24+ hours	1.01	0.97	1.05	0.95	1.07	0.41
7+ days	0.95	0.90	1.00	0.88	1.03	0.91
Concealed Carry						
Shall Issue	1.03	1.00	1.06	0.98	1.08	0.10
Permitless Carry	1.02	0.97	1.07	0.94	1.10	0.33
Firearm Suicides						
Background Checks						
Dealer BC	0.95	0.92	0.99	0.90	1.01	0.94
Universal BC	1.01	0.97	1.05	0.95	1.07	0.40
Minimum Age						
Possession 18+	0.97	0.93	1.02	0.91	1.04	0.80
Purchase 20+	0.96	0.92	1.00	0.90	1.02	0.89
Waiting Periods						
24+ hours	0.99	0.95	1.02	0.93	1.04	0.70
7+ days	0.99	0.95	1.04	0.92	1.07	0.59
Concealed Carry						
Shall Issue	1.03	1.00	1.06	0.99	1.07	0.09
Permitless Carry	1.01	0.96	1.06	0.94	1.09	0.41
Firearm Homicides						
Background Checks						
Dealer BC	1.00	0.94	1.07	0.91	1.11	0.48
Universal BC	1.07	1.00	1.14	0.96	1.18	0.10
Minimum Age						
Possession 18+	0.93	0.87	1.00	0.84	1.04	0.90
Purchase 20+	1.03	0.96	1.10	0.93	1.14	0.30
Waiting Periods						
24+ hours	1.04	0.98	1.11	0.96	1.14	0.17
7+ days	0.95	0.88	1.03	0.85	1.07	0.79
Concealed Carry						
Shall Issue	1.05	1.00	1.11	0.98	1.14	0.09
Permitless Carry	1.03	0.95	1.11	0.91	1.16	0.34

Note: IRR refers to incident rate ratios, CI to credible interval.

© 2024 Schell TL et al. *JAMA Network Open.*

eTable 5. Effect Sizes of State Laws on Firearm Death Rates in the Fifth Year After Implementation for Those Firearm Restrictions Implemented Nationally in 1994

Outcome	Posterior median IRR	80% CI Lower	80% CI Upper	95% CI Lower	95% CI Upper	Posterior probability of reduced deaths
Total Firearm Deaths	0.90	0.83	0.96	0.80	1.00	0.98
Firearm Suicides	0.91	0.85	0.97	0.82	1.00	0.97
Firearm Homicides	0.93	0.83	1.04	0.78	1.11	0.78

Note: These estimated combined effects are for state laws that mandated: (a) dealer background checks, (b) minimum age of possession 18+, and (c) one-day or greater waiting periods. These are not estimates of the effect of the 1994 federal restrictions on firearms, although the heterogeneity in these policies across states was affected by the 1994 Federal laws. IRR refers to incident rate ratios, CI to credible interval

© 2024 Schell TL et al. *JAMA Network Open.*

eTable 6. Effect Sizes of Law Classes on State Firearm Death Rates in the Fifth Year After Implementation Estimated Using Uninformative Priors

Outcome<br>Law Class	Posterior median IRR	80% CI Lower	80% CI Upper	95% CI Lower	95% CI Upper	Posterior probability of reduced deaths
Total Firearm Deaths						
Background Checks	1.03	0.97	1.10	0.94	1.13	0.26
Minimum Age	0.94	0.88	1.01	0.85	1.04	0.88
Waiting Periods	0.95	0.89	1.01	0.86	1.04	0.88
Child Access	0.93	0.89	0.97	0.87	0.99	0.99
Concealed Carry	1.05	0.97	1.12	0.94	1.16	0.21
Stand-your-ground	1.03	0.99	1.07	0.98	1.09	0.14
Purchase & Possess	0.92	0.84	1.00	0.80	1.05	0.89
Use & Storage	0.86	0.79	0.95	0.75	0.99	0.98
Most Restrictive	0.79	0.70	0.90	0.66	0.96	0.99
Firearm Suicides						
Background Checks	0.96	0.91	1.02	0.88	1.05	0.82
Minimum Age	0.93	0.87	0.99	0.84	1.02	0.93
Waiting Periods	0.98	0.93	1.03	0.90	1.06	0.69
Child Access	0.97	0.94	1.01	0.92	1.03	0.80
Concealed Carry	1.03	0.97	1.10	0.93	1.14	0.27
Stand-your-ground	1.02	0.99	1.05	0.97	1.07	0.23
Purchase & Possess	0.87	0.80	0.95	0.77	0.99	0.98
Use & Storage	0.93	0.85	1.01	0.81	1.05	0.88
Most Restrictive	0.81	0.72	0.91	0.68	0.97	0.99
Firearm Homicides						
Background Checks	1.14	1.02	1.27	0.96	1.35	0.07
Minimum Age	0.94	0.83	1.06	0.78	1.13	0.74
Waiting Periods	0.95	0.86	1.06	0.81	1.13	0.71
Child Access	0.87	0.81	0.94	0.78	0.98	0.99
Concealed Carry	1.06	0.92	1.21	0.86	1.30	0.29
Stand-your-ground	1.07	1.00	1.15	0.97	1.19	0.09
Purchase & Possess	1.02	0.87	1.19	0.80	1.30	0.43
Use & Storage	0.77	0.65	0.91	0.60	0.99	0.98
Most Restrictive	0.78	0.63	0.99	0.55	1.10	0.91

Note: The *Purchase & Possession* law class estimates the combined effect of restrictions on firearms through *Background Check*, *Minimum Age Requirement*, and *Waiting Period* laws; The *Use & Storage* class estimates the combined effect of restrictions through *Child Access*, *Concealed Carry*, and *Stand-your-ground* laws. The *Most Restrictive* class estimates the combined effect of restrictions on firearms through all six classes of laws studied. IRR refers to incident rate ratios, CI to credible interval

© 2024 Schell TL et al. *JAMA Network Open.*

eTable 7. Effect Sizes of Law Classes on State Firearm Death Rates in the Fifth Year After Implementation Estimated Without Additional Lagged Policy Spline

**Outcome**<br>**Law Class**	**Posterior median IRR**	**80% CI Lower**	**80% CI Upper**	**95% CI Lower**	**95% CI Upper**	**Posterior probability of reduced deaths**
Total Firearm Deaths						
Background Checks	1.01	0.96	1.07	0.93	1.10	0.38
Minimum Age	0.94	0.88	0.99	0.86	1.02	0.93
Waiting Periods	0.98	0.93	1.03	0.91	1.06	0.70
Child Access	0.95	0.91	0.98	0.89	1.00	0.97
Concealed Carry	1.05	0.99	1.12	0.96	1.16	0.14
Stand-your-ground	1.02	0.99	1.06	0.97	1.08	0.19
Purchase & Possess	0.93	0.86	1.00	0.82	1.05	0.89
Use & Storage	0.88	0.81	0.95	0.78	0.99	0.98
Most Restrictive	0.82	0.73	0.91	0.69	0.96	0.99
Firearm Suicides						
Background Checks	0.96	0.92	1.01	0.89	1.04	0.84
Minimum Age	0.93	0.89	0.99	0.86	1.01	0.95
Waiting Periods	0.98	0.94	1.03	0.92	1.05	0.70
Child Access	0.97	0.94	1.01	0.92	1.03	0.84
Concealed Carry	1.04	0.99	1.10	0.96	1.14	0.17
Stand-your-ground	1.02	0.99	1.05	0.97	1.07	0.23
Purchase & Possess	0.88	0.82	0.95	0.79	0.98	0.99
Use & Storage	0.92	0.85	0.99	0.82	1.02	0.94
Most Restrictive	0.81	0.74	0.89	0.70	0.94	1.00
Firearm Homicides						
Background Checks	1.04	0.96	1.13	0.91	1.19	0.27
Minimum Age	0.96	0.88	1.06	0.84	1.11	0.70
Waiting Periods	1.03	0.95	1.11	0.91	1.16	0.32
Child Access	0.92	0.87	0.98	0.84	1.01	0.96
Concealed Carry	1.09	0.99	1.20	0.94	1.26	0.13
Stand-your-ground	1.05	1.00	1.12	0.97	1.15	0.12
Purchase & Possess	1.03	0.91	1.17	0.85	1.25	0.37
Use & Storage	0.80	0.71	0.91	0.67	0.97	0.99
Most Restrictive	0.83	0.70	0.99	0.64	1.09	0.91

Note: The *Purchase & Possession* law class estimates the combined effect of restrictions on firearms through *Background Check*, *Minimum Age Requirement*, and *Waiting Period* laws; The *Use & Storage* class estimates the combined effect of restrictions through *Child Access*, *Concealed Carry*, and *Stand-your-ground* laws. The *Most Restrictive* class estimates the combined effect of restrictions on firearms through all six classes of laws studied. IRR refers to incident rate ratios, CI to credible interval

© 2024 Schell TL et al. *JAMA Network Open.*

eTable 8. Effect Sizes of Law Classes on Total Suicide and Total Homicide Death Rates in the Fifth Year After Implementation

Outcome<br>Law Class	Posterior median IRR	80% CI Lower	80% CI Upper	95% CI Lower	95% CI Upper	Posterior probability of reduced deaths
Total Suicides						
Background Checks	0.99	0.95	1.03	0.93	1.05	0.67
Minimum Age	1.00	0.96	1.05	0.93	1.08	0.48
Waiting Periods	0.96	0.92	1.00	0.90	1.02	0.92
Child Access	0.98	0.95	1.01	0.94	1.02	0.82
Concealed Carry	1.00	0.96	1.05	0.93	1.08	0.45
Stand-your-ground	1.01	0.98	1.03	0.97	1.05	0.37
Purchase & Possess	0.95	0.89	1.01	0.86	1.04	0.88
Use & Storage	0.97	0.91	1.03	0.88	1.07	0.74
Most Restrictive	0.92	0.85	1.00	0.81	1.04	0.90
Total Homicides						
Background Checks	1.05	0.97	1.13	0.93	1.18	0.21
Minimum Age	0.98	0.91	1.07	0.87	1.11	0.60
Waiting Periods	1.00	0.93	1.08	0.89	1.12	0.51
Child Access	0.94	0.89	0.99	0.87	1.01	0.95
Concealed Carry	1.03	0.95	1.12	0.91	1.17	0.31
Stand-your-ground	1.03	0.99	1.09	0.96	1.11	0.18
Purchase & Possess	1.03	0.92	1.15	0.87	1.23	0.36
Use & Storage	0.88	0.79	0.98	0.75	1.04	0.94
Most Restrictive	0.91	0.78	1.06	0.72	1.15	0.79

Note: The *Purchase & Possession* law class estimates the combined effect of restrictions on firearms through *Background Check*, *Minimum Age Requirement*, and *Waiting Period* laws; The *Use & Storage* class estimates the combined effect of restrictions through *Child Access*, *Concealed Carry*, and *Stand-your-ground* laws. The *Most Restrictive* class estimates the combined effect of restrictions on firearms through all six classes of laws studied. IRR refers to incident rate ratios, CI to credible interval

© 2024 Schell TL et al. *JAMA Network Open.*

eTable 9. Separate Effect Estimates for Each Policy on Total Firearm Deaths using the Callaway and Sant'Anna (2021) Staggered DiD Method

**Law Class**<br>**Law Subclass**	**Policy transitions used**	**IRR**	**95% LCI**	**95% UCI**
Background Checks				
Dealer BC	8	0.97	0.87	1.07
Universal BC	14	0.99	0.93	1.06
Minimum Age				
Possession 18+	20	0.99	0.96	1.03
Purchase 20+	13	0.99	0.95	1.03
Waiting Periods				
24+ hours	3	0.98	0.87	1.09
7+ days	6	0.97	0.90	1.03
Child Access	19	0.95	0.86	1.03
Concealed Carry				
Shall Issue	36	1.01	0.93	1.09
Permitless Carry	14	1.04	0.96	1.12
Stand-your-ground	28	1.07	1.00	1.14

Note: IRR refers to incident rate ratios, CI to credible interval

© 2024 Schell TL et al. *JAMA Network Open.*

eFigure 1. Effect Estimates of 6 Classes of Firearm Regulations Over Time, by Type of Firearm Death

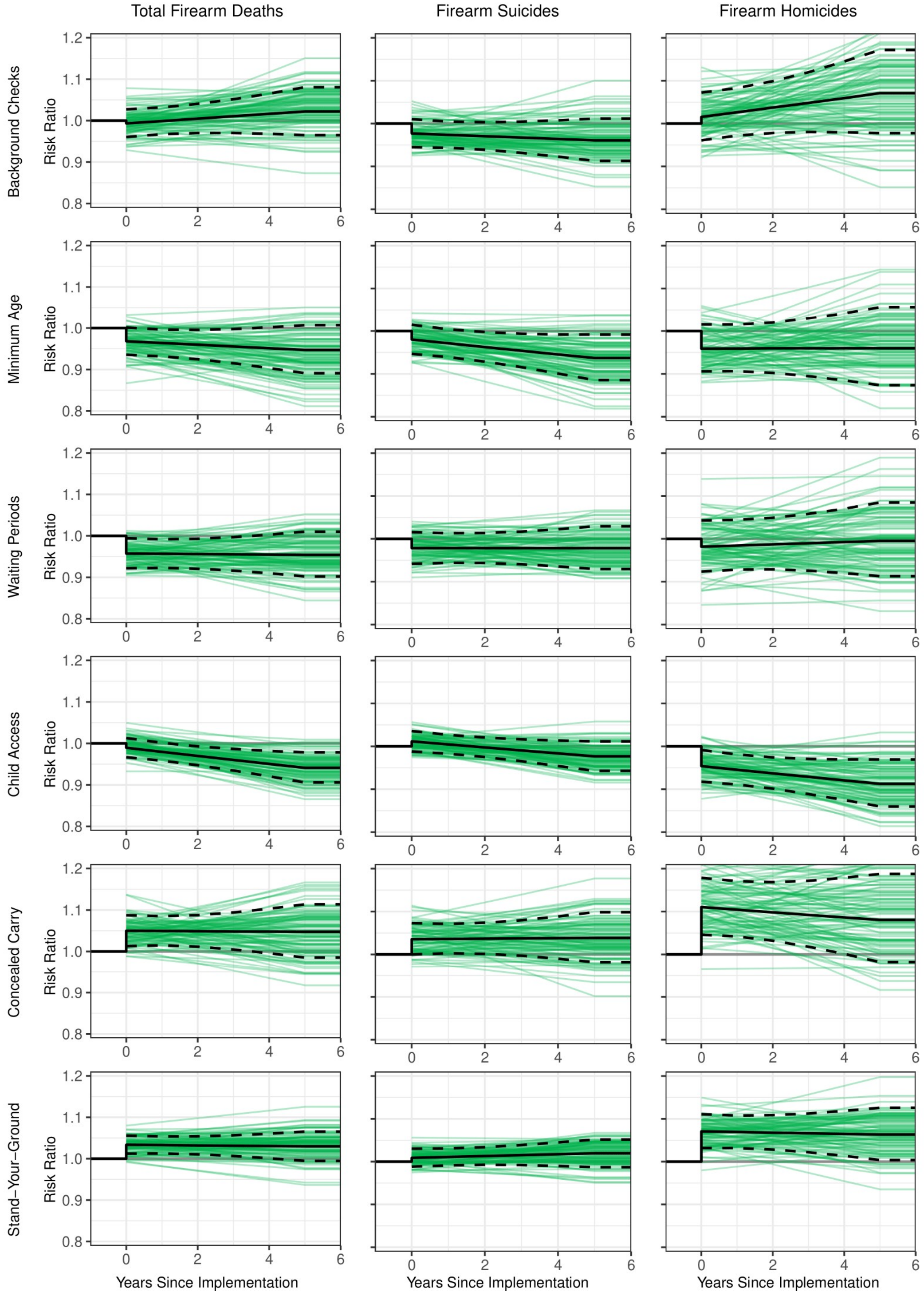

The posterior median, 80% credible interval, and 100 samples from the posterior distribution are plotted.

© 2024 Schell TL et al. *JAMA Network Open.*

eFigure 2. Effect Estimates of Combinations of Firearm Regulations Over Time, by Type of Firearm Death

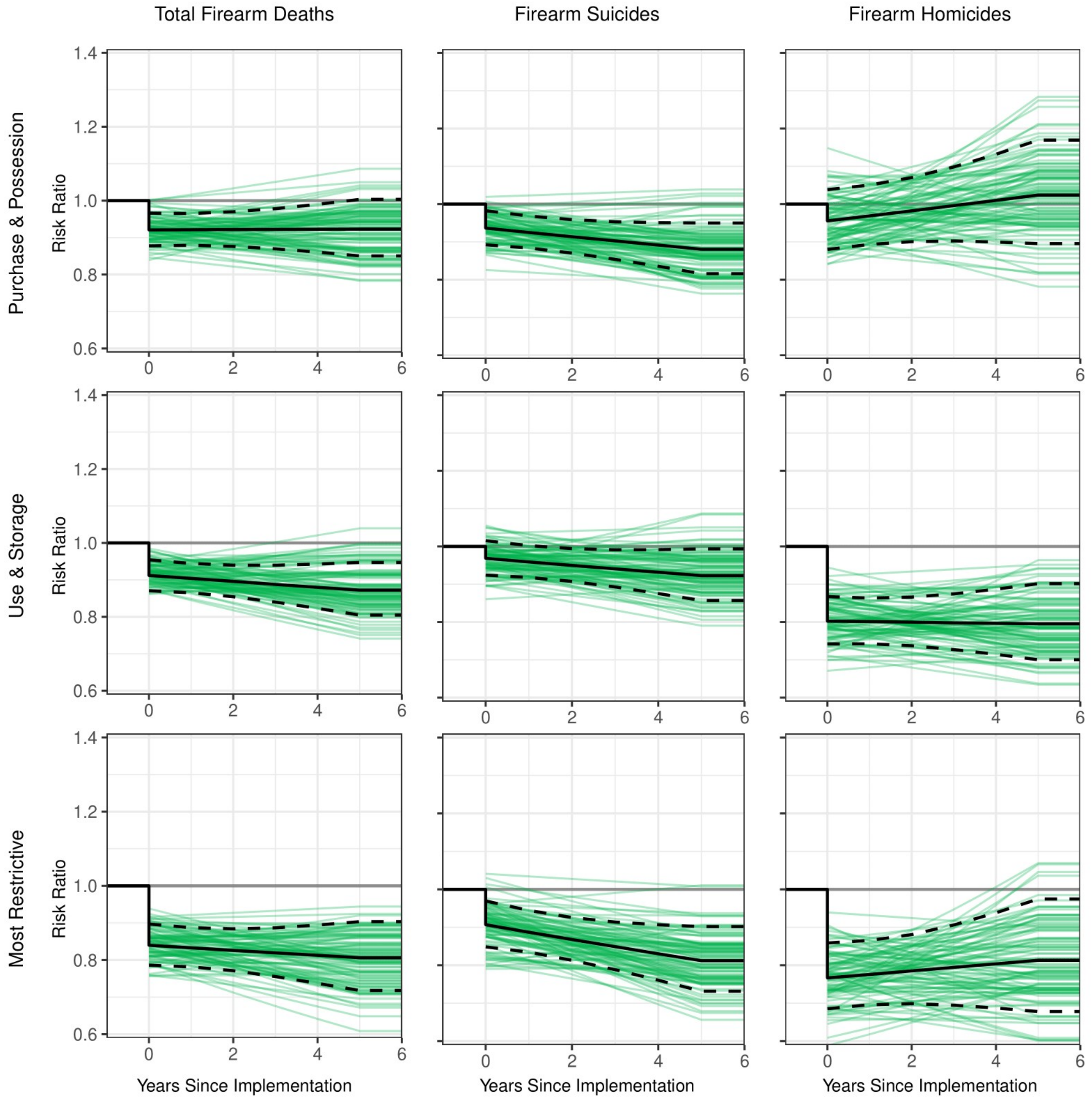

The posterior median, 80% credible interval, and 100 samples from the posterior distribution are plotted.

© 2024 Schell TL et al. *JAMA Network Open.*

eReferences

Alvaredo F, Atkinson AB, Piketty T, Saez E, Zucman G. The World Inequality Database. 2020. http://www.wid.world/.

Ben-Michael, Eli, Avi Feller, and Elizabeth A. Stuart. "A trial emulation approach for policy evaluations with group-level longitudinal data." *Epidemiology* (Cambridge, Mass.) 32, no. 4 (2021): 533.

Braga AA, Griffiths E, Sheppard K, Douglas S. Firearm instrumentality: Do guns make violent situations more lethal? *Annual Review of Criminology* 2021;4:147-164.

Callaway, Brantly, and Pedro HC Sant'Anna. "Difference-in-differences with multiple time periods." *Journal of econometrics* 225, no. 2 (2021): 200-230.

Cefalu M, Schell TL, Griffin BA, Smart R, Morral AR. Estimating effects within nonlinear autoregressive models: A case study on the impact of child access prevention laws on firearm mortality. *arXiv preprint arXiv:2109.03225*, 2021.

Cherney S, Morral AR, Schell TL, Smucker S. *Development of the RAND State Firearm Law Database and supporting materials.* Santa Monica, CA: RAND Corporation, 2020. Available as of June 14, 2023: https://www.rand.org/pubs/tools/TLA243-2-v2.html

Gelman A, Hwang J, Vehtari A. Understanding predictive information criteria for Bayesian models. *Statistics and Computing* 2014; 24:997-1016.

Hernán, Miguel A. Methods of Public Health Research — Strengthening Causal Inference from Observational Data. *New England Journal of Medicine*. 2021, 385(15):1345 10.1056/NEJMp2113319

Pearl J. Causality: *Models, reasoning and inference*. 2nd ed., London, UK: Cambridge University Press, 2009.

Schell, TL., Cefalu, M., Griffin BA, Smart, R. and Morral, AR. Changes in firearm mortality following the implementation of state laws regulating firearm access and use. *Proceedings of the National Academy of Sciences*, 2020b 117(26), 14906-14910. PMCID: PMC7334522

Schell TL, Griffin BA, Morral AR. *Evaluating methods to estimate the effect of state laws on firearm deaths*. Santa Monica, CA: RAND Corporation, 2018.

Schell TL, Peterson S, Vegetabile BG, Scherling A, Smart R, Morral AR. *State-level estimates of household firearm ownership*. Santa Monica, CA: RAND, 2020a.

Smart R, Morral AR, Schell TL. *The magnitude and sources of disagreement among gun policy experts, second edition*. Santa Monica, CA: RAND Corporation, 2021.

Smart R, Schell TL, Cefalu M, Morral AR. Impact on nonfirearm deaths of firearm laws affecting firearm deaths: A systematic review and meta-analysis. *American Journal of Public Health* 2020b;110(10): e1-e9.

© 2024 Schell TL et al. *JAMA Network Open.*
