# Supplementary material for: State Policies Regulating Firearms and Changes in Firearm Mortality
Source: JAMA Netw Open. 2024 Jul 31;7(7):e2422948. doi: 10.1001/jamanetworkopen.2024.22948 (PMC11292452; doi:10.1001/jamanetworkopen.2024.22948)
Supplement: Supplement 2. — Data Sharing Statement [file jamanetwopen-e2422948-s002.pdf]

Data Sharing Statement

Schell. State Policies Regulating Firearms and Changes in Firearm Mortality. *JAMA Netw Open*. Published July 31, 2024. doi:10.1001/jamanetworkopen.2024.22948

Data
Data available: Yes
Data types: Other (please specify)
Additional Information: A small portion of the data cannot be released under our DUA with CDC. All of the remaining data has been made available.
How to access data: https://osf.io/pae7s
When available: With publication

Supporting Documents
Document types: Statistical/analytic code
How to access documents: https://osf.io/pae7s
When available: With publication

Additional Information
Who can access the data: anyone requesting the data
Types of analyses: for any purpose
Mechanisms of data availability: public download without investigator approval
